# Change in Auxin and Cytokinin Levels Coincides with Altered Expression of Branching Genes during Axillary Bud Outgrowth in Chrysanthemum

**DOI:** 10.1371/journal.pone.0161732

**Published:** 2016-08-24

**Authors:** Robrecht Dierck, Ellen De Keyser, Jan De Riek, Emmy Dhooghe, Johan Van Huylenbroeck, Els Prinsen, Dominique Van Der Straeten

**Affiliations:** 1 Institute for Agricultural and Fisheries Research (ILVO), Caritasstraat 39, Melle, Belgium; 2 University of Antwerp, Groenenborgerlaan 171, Antwerp, Belgium; 3 Laboratory of Functional Plant Biology, Department of Physiology, Ghent University, K.L. Ledeganckstraat 35, Ghent, Belgium; Ecole Normale Superieure, FRANCE

## Abstract

In the production and breeding of *Chrysanthemum sp*., shoot branching is an important quality aspect as the outgrowth of axillary buds determines the final plant shape. Bud outgrowth is mainly controlled by apical dominance and the crosstalk between the plant hormones auxin, cytokinin and strigolactone. In this work the hormonal and genetic regulation of axillary bud outgrowth was studied in two differently branching cut flower *Chrysanthemum morifolium* (Ramat) genotypes. C17 is a split-type which forms an inflorescence meristem after a certain vegetative period, while C18 remains vegetative under long day conditions. Plant growth of both genotypes was monitored during 5 subsequent weeks starting one week before flower initiation occurred in C17. Axillary bud outgrowth was measured weekly and samples of shoot apex, stem and axillary buds were taken during the first two weeks. We combined auxin and cytokinin measurements by UPLC-MS/MS with RT-qPCR expression analysis of genes involved in shoot branching regulation pathways in chrysanthemum. These included bud development genes (*CmBRC1*, *CmDRM1*, *CmSTM*, *CmLsL*), auxin pathway genes (*CmPIN1*, *CmTIR3*, *CmTIR1*, *CmAXR1*, *CmAXR6*, *CmAXR2*, *CmIAA16*, *CmIAA12*), cytokinin pathway genes (*CmIPT3*, *CmHK3*, *CmRR1*) and strigolactone genes (*CmMAX1* and *CmMAX2*). Genotype C17 showed a release from apical dominance after floral transition coinciding with decreased auxin and increased cytokinin levels in the subapical axillary buds. As opposed to C17, C18 maintained strong apical dominance with vegetative growth throughout the experiment. Here high auxin levels and decreasing cytokinin levels in axillary buds and stem were measured. A differential expression of several branching genes accompanied the different hormonal change and bud outgrowth in C17 and C18. This was clear for the strigolactone biosynthesis gene *CmMAX1*, the transcription factor *CmBRC1* and the dormancy associated gene *CmDRM1*, that all showed a decreased expression in C17 at floral transition and an increased expression in C18 with continuous vegetative growth. These results offer a case study for Chrysanthemum, showing an altered cytokinin to auxin balance and differential gene expression between vegetative growth with apical dominance and transition to generative growth with loss of apical dominance and axillary bud outgrowth. This suggests a conservation of several aspects of the hormonal and genetical regulation of bud outgrowth in Chrysanthemum. Furthermore, 15 previously uncharacterised genes in chrysanthemum, were described in this study. Of those genes involved in axillary bud outgrowth we identified *CmDRM1*, *CmBRC1 and CmMAX1* as having an altered expression preceding axillary bud outgrowth, which could be useful as markers for bud activity.

## Introduction

*Chrysanthemum morifolium* (Ramat), called the florist’s chrysanthemum, is an economically important horticultural crop. Chrysanthemums show a great variation in sizes and shapes: garden and potted plants are highly branching, while cut flowers frequently show limited branching and sometimes even removal of axillary buds is required to obtain single flowered stems. Modification of plant architecture through shoot branching is a relevant factor in breeding and production of chrysanthemum. Shoot branching or axillary bud outgrowth in herbaceous shoots is regulated by a complex interaction of external factors (light, temperature, nutrients and pruning) and plant hormone signalling [1–3]. The most prominent hormones associated with the regulation of bud outgrowth are auxins, strigolactones and cytokinins [3]. Auxins are widely regarded to be responsible for apical dominance, the phenomenon where the growth of the vegetative shoot apex exerts a control over the outgrowth of axillary buds [4]. Removal of the shoot apex and floral transition, releases the apical control over lateral bud outgrowth [5]. Auxin regulation of apical dominance can be explained by the young expanding leaves and the shoot apex that produce auxin, which is transported through the stem towards the roots in a polar auxin transport stream facilitated largely by the auxin transport protein PIN1 [6,7] in the basal membranes of xylem parenchyma cells. On its way to the roots the auxin exerts an inhibition of the axillary bud outgrowth.

Since the basipetal transport does not deliver auxin into the axillary buds directly, an indirect action of auxin is suggested [8]. In literature the indirect inhibition by auxin is explained by two non-mutually exclusive models: the second messenger model [9] and the canalisation model [10].

The canalisation model explains the inhibition of axillary bud outgrowth by the polar auxin stream in the stem that acts as an auxin sink. The shoot apex and the axillary buds are auxin sources that compete with each other for the ability to export auxin to the sink. Evidence for this model comes from the observations in Arabidopsis that strigolactones inhibit axillary bud outgrowth by reducing PIN1 mobilisation, as such restricting polar auxin transport [11,12].

The second messenger model states that a signal downstream of auxin is responsible for the inhibition of bud outgrowth. Both cytokinins and strigolactones control shoot branching downstream of auxins, and thus may be considered as secondary messengers. Cytokinins have a positive effect on the outgrowth of axillary buds. This is supported by observations in pea of stimulation of bud outgrowth upon exogenous application of cytokinins [13] and increasing cytokinin biosynthesis in stems and axillary buds at the time of outgrowth of axillary buds [14]. As a response to auxin signalling, the biosynthesis of cytokinins is inhibited in Arabidopsis and pea [15,16], while its degradation is promoted in pea [16]. Like auxins, strigolactones inhibit axillary bud outgrowth, which was shown in Arabidopsis, rice and pea [17,18] and the biosynthesis of strigolactones is upregulated by auxin in Arabidopsis and pea [19,20].

**Fig 1:**
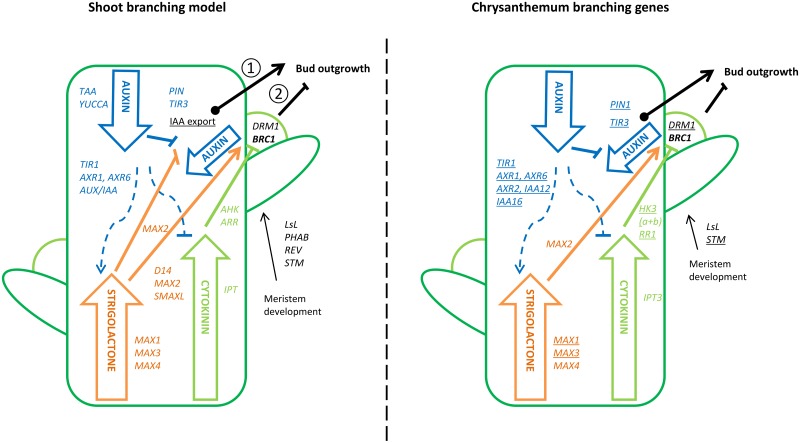
Key shoot branching regulatory pathways and involvement of the branching genes used in this study of axillary bud outgrowth. The left panel shows the shoot branching model from what is known in other species, while the right panel shows the branching genes that have been described in chrysanthemum with the genes that were isolated in this study underlined. ① indicates the bud outgrowth regulation that follows the auxin canalisation model where auxin export is required for an axillary bud to grow out. ② indicates the regulation of bud outgrowth according to the second messenger model where auxin indirectly regulates bud outgrowth through strigolactone and cytokinin signalling.

In this way the physiological regulation of shoot branching involves the activity of many genes involved in the local axillary meristem maintenance and in the pathways of auxin, cytokinin and strigolactones (Fig 1). The formation of axillary meristems in Arabidopsis, involves the lateral suppressor gene *LAS* [21], *REVOLUTA (REV)* and *PHABULOSA (PHAB) [22]*. *SHOOT MERISTEMLESS (STM)* is another gene involved in the formation of axillary meristems [23] and can be used as an early marker for axillary meristem initiation [24]. In *Euphorbia esula*, dormant adventitious buds were shown to have upregulated *STM* expression after defoliation treatment to induce bud growth [25].

A central regulator of axillary bud outgrowth is the transcription factor *BRC1* (*BRANCHED1*) that is expressed locally in the axillary bud and inhibits outgrowth [26]. Many of the regulatory pathways affect shoot branching by acting on the expression of *BRC1*. In Arabidopsis and pea, strigolactones promote *BRC1* expression while cytokinins inhibit *BRC1* expression [27,28]. *BRC1* is also involved in the floral transition as it is under control of the florigen pathway with a proposed interaction between *FT* (*FLOWERING LOCUS T*) and *BRC1* whereby *BRC1* is inactivated, promoting branching at floral transition [29]. A dormancy marker similar to *BRC1* is *DRM1* (*DORMANCY ASSOCIATED PROTEIN 1*). *DRM1* expression was shown to decrease in pea [30] and Arabidopsis [31] after the release of dormancy by decapitation. In wheat and sorghum, high expression of *DRM1* and *BRC1* have been reported in dormant axillary buds [32,33].

Auxin (IAA) biosynthesis in Arabidopsis involves the activity of the *TAA* (*TRYPTOPHAN AMINOTRANSFERASE*) and *YUCCA* genes [34]. For auxin transport *TIR3* (*TRANSPORT INHIBITOR RESPONSE 3*) encodes a protein that is required in the mobilization of PIN1 proteins in the cell membrane [35]. *Tir3* mutant plants showed reduced auxin transport and increased shoot branching in Arabidopsis [36]. Auxin signalling involves TIR1 (TRANSPORT INHIBITOR RESPONSE 1), AXR1 (AUXIN RESISTANT 1) and AXR6 (AUXIN RESISTANT 6)that are required to form an F-box complex for ubiquitination of Aux/IAA repressors [37,38] leading to activation of auxin response genes [39]. *AXR1* was shown to be responsible for the auxin induced inhibition of axillary bud growth in Arabidopsis [40] and is required for the upregulation of strigolactone biosynthesis and downregulation of cytokinin biosynthesis [20]. Aux/IAA proteins are degraded by the TIR1 F-box complex as a response to auxin [41–43]. The expression of these Aux/IAA genes is upregulated by auxin and is useful as a marker for auxin signalling [44,45].

Strigolactone regulation of shoot branching involves the *MORE AXILLARY GROWTH* (*MAX*) genes in Arabidopsis and it’s homologues *RAMOSUS* (*RMS*), *DECREASED APICAL DOMINANCE* (*DAD*) and *DWARF* (*D*) in pea, petunia and rice respectively. *MAX3* and *MAX4* convert carotenoid precursors and *MAX1* that is involved in the final step of strigolactone biosynthesis *[8,46,47]*. Strigolactone signalling involves an F-Box complex with MAX2 and DWARF14 (D14), allowing the degradation of SMAX1-like proteins to enable strigolactone response [48,49].

For cytokinin biosynthesis *IPT* (*ISOPENTENYLTRANSFERASE*) is the rate limiting step in the pathway [50]. In Arabidopsis, the cytokinin receptors are histidine kinase (HK) proteins that include HK4, *HK3* and *HK2*. These cytokinin receptors relay a phosphor signal to RESPONSE REGULATOR proteins (RR) that act as transcription factors for downstream cytokinin responses [51]. Type A *RR* genes have been shown to be required for axillary bud activation by cytokinin [52].

In chrysanthemum only few genes involved in shoot branching have been isolated (Fig 1). *CmBRC1*’s activity has been described in chrysanthemum with high expression in inhibited axillary buds and downregulation when buds were activated [53]. A *LATERAL SUPPRESSOR LIKE* (*LsL*) gene was isolated in chrysanthemum [54] and plants transformed with the antisense *LsL* gene showed reduced branching and increased IAA content in the shoot tip while transformation with a sense *LsL* construct showed increased branching and reduced IAA content in the shoot tip [55]. The strigolactone biosynthesis gene *CmMAX3* showed upregulated expression in plants with inhibited axillary buds by phosphorous (Pi) starvation [56]. *CmMAX4* was studied in the strigolactone regulation of shoot branching and its expression was reported to be upregulated by exogenous auxin treatment [57]. *CmMAX2* was found to be expressed in axillary buds and stems and is necessary for the response to strigolactone, including inhibition of shoot branching *[58]*. Overexpression of the *CmIPT3* gene in chrysanthemum resulted in enhanced branching [59].

Aside from looking at hormone levels directly, the expression of these genes is interesting as a potential indicator of axillary bud activity. Because of the limited number of genes available in chrysanthemum, several key genes involved in the shoot branching pathways were isolated in this study and their gene expression levels were determined during branching. Two distinctive chrysanthemum genotypes with different branching patterns were analysed. In the first genotype, C17, the transition from vegetative to generative growth released apical dominance, while the second one, C18, remained vegetative. Axillary bud outgrowth measurements were related to levels of auxin and cytokinin in apex, stem and axillary bud samples in order to investigate the hormonal regulation during axillary bud outgrowth. The expression of candidate branching genes was analysed in the same samples to show the underlying transcriptional control during bud growth.

## Materials and Methods

### Plant Material and Growth Conditions

**Fig 2:**
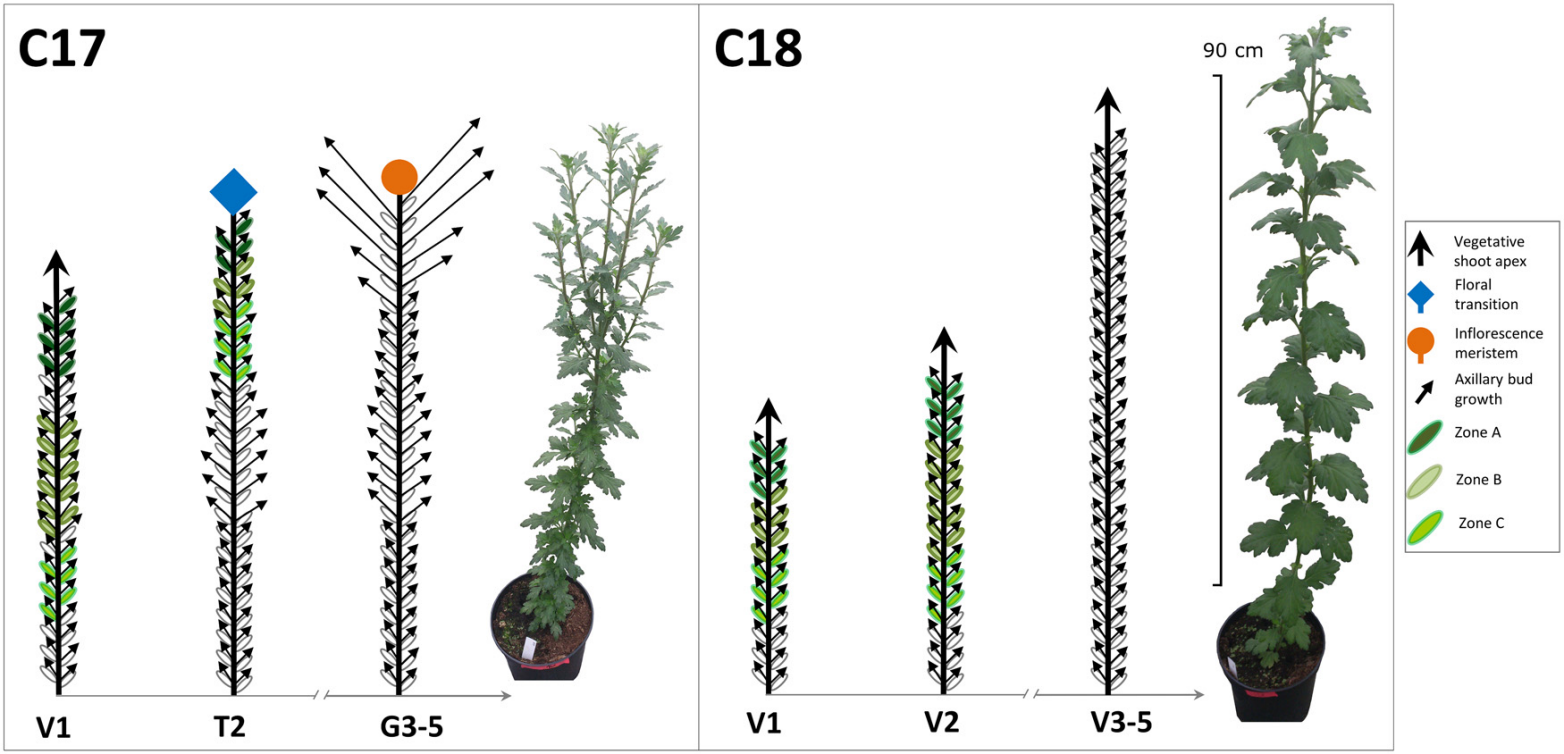
Branching phenotypes of the cut flower chrysanthemum C17 and C18. C17 grows vegetatively in week 1 (V1) and starts floral transition in week 2 (T2) after which it shows generative growth and outgrowth of subapical axillary buds (G3-G5). C18 shows vegetative growth throughout week 1–5 (V1 to V5). Sampling zones A, B, and C are indicated in shades of green.

*Chrysanthemum morifolium* (Ramat) cut flower genotypes C17 and C18, provided by Dekker Chrysanten BV., The Netherlands) were used in this study. Both genotypes are commercial cultivars that have an unrelated genetical background in their breeding history. C17 is a split type chrysanthemum, forming an inflorescence meristem at the shoot apex once a number of vegetative leaves are produced. This results in a release from apical dominance with outgrowth of subapical axillary buds. C18 is a non-split type with vegetative growth during long day (LD) conditions and generative growth under short day (SD) conditions. A diagram of the growth habit of both genotypes under LD is shown in Fig 2. Of each genotype 100 rooted cuttings were potted into 3L pots in standard peat substrate. Plants were grown at 20 cm spacing in a standard greenhouse under natural LD conditions and average day/night temperature of 20 ± 2°C.

### Plant Morphological Measurements

The start of measurements and sampling was in the first week of July 2014, 2 weeks after potting. The total plant height and length of axillary buds/shoots was measured for 10 plants of each genotype during 5 consecutive weeks, referred to as: V1, T2, G3, G4, G5 (C17) or V1, V2, V3, V4, V5 (C18) with vegetative growth indicated by V, generative growth by G and the transition to generative growth indicated by T. The timing of floral transition in C17 was known from previous bud outgrowth experiments (data not shown).

### Sampling Plant Material

In total, 90 plants were sampled per genotype at the first two time points (V1 and T2 for C17, V1 and V2 for C18). Three replicates were sampled per time point, each replicate consisting of 15 plants. For C17 at V1, the shoot apex (the top part of the shoot above the first fully unfolded leaf) and the 30 nodal positions under the apex were sampled. At T2, the apex and 15 nodal positions under the apex were sampled, as the lateral buds below this zone had already grown out. For C18 at V1, the shoot apex and 22 underlying nodal positions were harvested, while at V2, the apex and 27 subapical nodal positions were taken. For each nodal position, the axillary buds and stems were sampled separately. Samples were frozen in liquid nitrogen and stored at -80°C. Before analysis, the samples were pooled based on the pattern of axillary bud outgrowth until week 5. They were subdivided to represent vertical positions along the stem that are at different distances and influences from apical dominance. From top to bottom these were named Apex, Zone A, Zone B and Zone C (Fig 2 and Table 1). Pooled samples were ground in liquid nitrogen and separate samples were weighed for gene expression analysis (100mg/sample) and UPLC-MS/MS (50mg/sample).

**Table 1:** Different zones and the corresponding nodal position from which stem and axillary bud samples were harvested in Chrysanthemum C17 and C18. For C17 in V1 nodal positions 37 to 31 (Zone A V1) were pooled together to represent inhibited axillary buds under apical dominance. Positions 26 to 16 in week 1 (Zone B V1) contain axillary buds that are further away from the shoot apex and show more outgrowth. Positions 13 to 8 (Zone C V1) represent bottom axillary buds inhibited by correlative inhibition of middle axillary buds. In T2 nodal positions 45 to 42 (Zone A T2) were pooled because the buds in this section showed the strongest outgrowth after T2 and release from apical dominance. Nodal positions 41 to 38 (Zone B T2) were pooled because these buds showed a diminishing outgrowth after week 2 when compared to the nodal positions above them. Nodal positions 37 to 31 (Zone C T2) were pooled to represent buds that show inhibited outgrowth after week 2. For C18 in V1 nodal positions 24–20 represent subapical inhibited buds (Zone A V1), positions 19–14 represent buds that are less inhibited by apical dominance (Zone B V1) and positions 13–8 represent buds furthest away from apical influence. In V2 positions 30–25 represent inhibited subapical buds (Zone A V2), positions 24–20 (Zone B’ V2) and 19–14 (Zone B” V2) represent less inhibited buds with zone B’ and B” the same positions as zone A and B in V1.

Sample zone	Position and developmental status	C17		C18	
		V1	T2	V1	V2
**Shoot apex**	Shoot apical meristem.				
**Zone A**	Nodal positions directly under the shoot apex and under apical dominance control. For C17 these are the positions that show release from apical dominance and strongest bud outgrowth after floral transition in T2.	37–31	45–42	24–20	30–25
**Zone B**	Nodal positions further away from the shoot apex and apical dominance. For C17 T2 these positions show weaker bud outgrowth after floral transition in T2. For C18 V2 zone B was subdivided in 2 pools. Pool B’ are the same positons as zone A from V1. Pool B” are the positions from zone B V1.	26–16	41–38	19–14	B’ 24–20
					B” 19–14
**Zone C**	Nodal positions furthest away from the shoot apex and apical dominance. For C17 T2 these are positions that show inhibited bud outgrowth after floral transition in T2.	13–8	37–31	13–8	13–8

### Hormone Measurement

Following a solid phase extraction, samples were analysed with UPLC-MS/MS (Acquity TQD) for quantification of free IAA and total cytokinins (the total of individual measurements of DHZR, trans-ZR, cis-ZR, DHZ, trans-Z, cis-Z, DH-ZNG, ZNG, IP-G, iPA, iP, MS-iP, MS-iPA, BAP, oT, pT, mT, MeOT, MemT, BAR, oTR, mTR pTR, BA3G, BA7G, BA9G, MeoTR, MemTR, oT9G, mT9G, pT9G, MemT9G) as described by Prinsen et al. [60,61].

### Gene Isolation

For *CmMAX1* degenerate primers were developed based on MAX1 amino acid sequences from Arabidopsis, tomato, poplar, soy, *Medicago*, vine and *Ricinus* S1 Table. Furthermore, a BLAST search was done to the chrysanthemum transcriptome database of Xu et al. [62] using Arabidopsis genes known to be involved in branching (corresponding accession numbers are indicated in Table S.2). This resulted in orthologous sequences for *CmMAX3*, *CmDRM1*, *CmSTM*, *CmRR1*, *CmHK3a*, *CmHK3b*, *CmAXR1*, *CmAXR2*, *CmIAA16*, *CmAXR6*, *CmIAA12*, *CmPIN1*, *CmTIR1* and *CmTIR3*. Reference genes were identified by BLAST against the NCBI database. These included *CmACT2*, *CmATUB*, *CmUBQ10*, *CmUBC*, *CmEF1α*, *CmCACS*, *CmEXP5*, *CmEXP6*, *CmPGK*, *CmPSAA* and *CmHH3*. Sequences of *CmBTUB_*AB608732 and *CmMTP_*AB542716.1. were available for Chrysanthemum. Primers S2 Table. were developed using Primer3 PLUS. Genes were cloned using a pGEM-T kit (Promega) and sequenced according to the protocol of the Big Dye Terminator Cycle Sequencing kit version 1.1 on an ABI Prism 3130 *xl* Genetic analyser (Applied Biosystems). BlastX [63] was used to validate isolated fragment identity.

### Gene Expression Analysis

RNA was extracted with a modified CTAB protocol (Luypaert et al., submitted). RNA concentrationand qualitywas verified with the NanoDrop spectrophotometer S3 Table. (Isogen Life Sciences) and Experion^™^ automated electrophoresis S1 Fig. using the StdSense analysis kit (Bio-Rad) was performed on a subset of samples as described in De Keyser et al. [64]. RNA was treated with *DNase* I according to manufacturer’s protocol (*DNA-free*^™^, Ambion). DNase treated RNA (800ng) was converted to cDNA with the iScript cDNA Synthesis kit (Bio-Rad). The no reverse-transcriptase controls (noRTs) were made using a mixture of DNase treated RNA and nuclease free water (1:1). cDNA and noRT samples were diluted (1:5) prior to use. Expression of branching genes was quantified using the LightCycler^®^ 480 (Roche). In white plates (Bio-Rad) a final volume of 10 μl, containing 2 μl cDNA, 300nM of both primers, and 5 μl of SensiFAST^™^ SYBR^®^ No-ROX (Bioline) was added. PCR amplification and melting curve analysis was done as described in De Keyser et al. [64]. Primer sequences are listed in S4 Table.; besides the isolated genes also genes already known to be involved in axillary bud outgrowth in chrysanthemum were used: *CmBRC1* [53], *CmLSL* [54], *CmIPT3* (Acc. Nr. JQ711176.1) and *CmMAX2* [58]. NTCs (no template controls) and noRTs were included for all genes; technical replicates were omitted. All stem or apex/bud samples, respectively, were analysed on the same plate for every gene (sample maximisation method). Due to limitations in the amount of cDNA available, the RT-qPCR analysis was done on two separate batches of cDNA (same RNA but 2 individual cDNA synthesis steps). With the first batch, GeNorm [65] analysis was done and the expression of *CmBRC1*, *CmIPT*, *CmLsL*, *CmMAX1* and *CmMAX2* was quantified; the second batch was used for expression analysis of *CmRR1*, *CmAXR1*, *CmAXR2*, *CmAXR6*, *CmHK3a*, *CmHK3b*, *CmDRM1*, *CmIAA12*, *CmIAA16*, *CmMAX3*, *CmPIN1*, *CmSTM*, *CmTIR1* and *CmTIR3*; reference genes were analysed again on these cDNA samples as well. For all gene expression data analysis, qbase^+^ software (Biogazelle) [66] was used. In case the difference between the Cq of noRT and samples was smaller as 5, samples were excluded from further analysis. Mean gene-specific amplification efficiencies S5 Table. were determined using LinRegPCR [67,68]. Gene expression results are presented as Calibrated Normalized Relative Quantities, normalised using 3 validated reference genes for stem and apex/bud samples S6 Table.; different normalisation factors were used for batch 1 and 2. All further analysis was done in MS Excel.

### Statistical Analysis

Kruskal-Wallis one-way analysis of variance and Spearman correlation tests were performed with SPSS statistical software (SPSS 22, IBM Corp. Released 2014. IBM SPSS Statistics for Windows, Version 22.0. Armonk, NY: IBM Corp.). Also in SPSS, UPGMA hierarchical clustering of the genes was done based on a Pearson correlation matrix. Discriminant analysis was performed in order to discriminate the global gene expression between tissues and time points. For statistical analysis of gene expression the log-transformed CNRQ values were used.

## Results

### C17 Initiates Axillary Bud Outgrowth at Floral Transition and C18 Shows Continuous Vegetative Growth and Apical Dominance

Two different genotypes were used for branching analysis. Genotype C17 shifted from vegetative growth with apical dominance (V1) to generative growth with loss of apical dominance at T2 (floral transition) and consecutive outgrowth of axillary buds (G3-G5). Genotype C18 showed vegetative growth throughout all time points V1-V5. (Fig 2).

**Fig 3:**
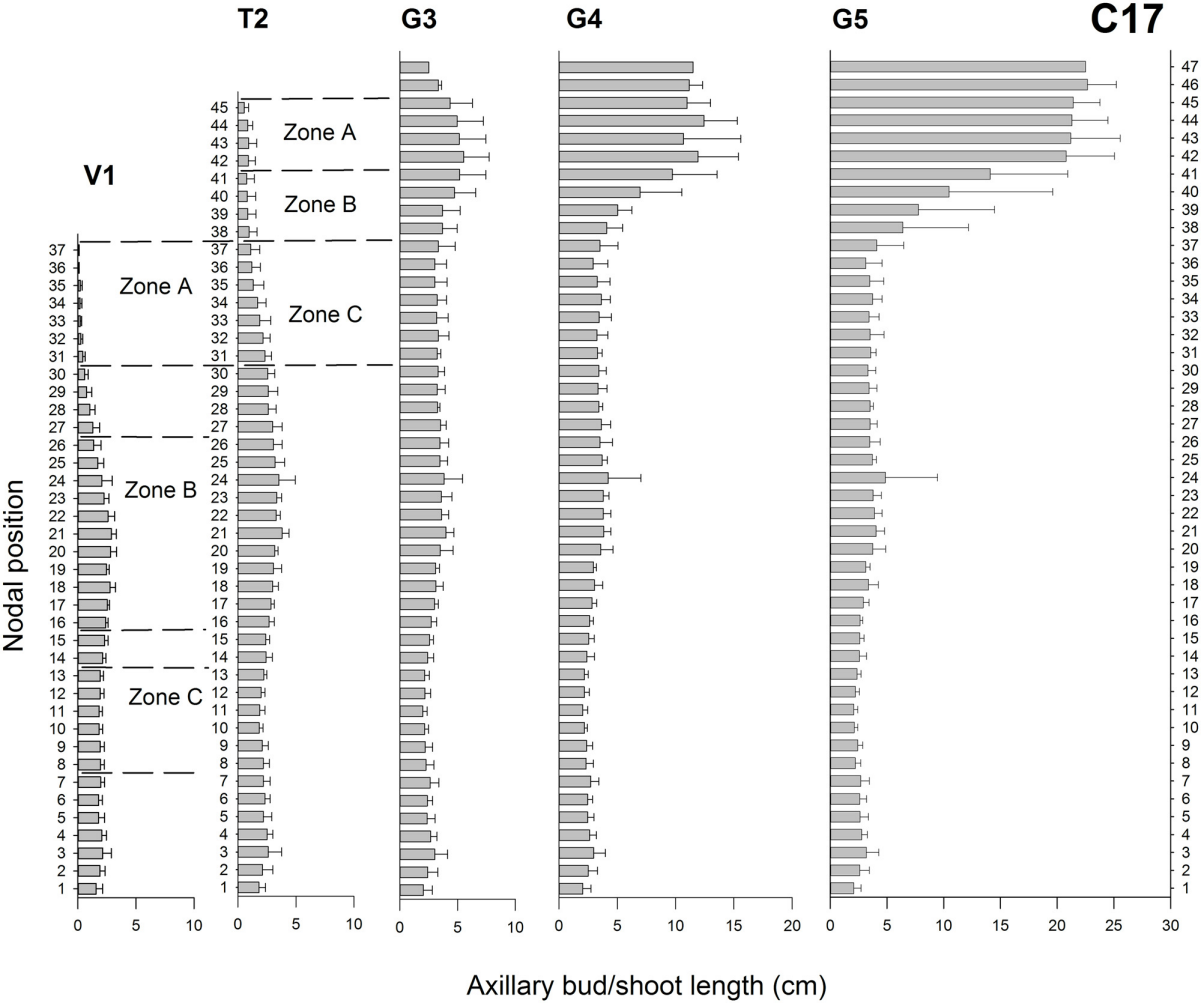
Axillary bud lengths for chrysanthemum C17 in V1, T2 and G3 to G5. Mean bud/shoot length (±SE; n = 10) is presented on the x-axis for every nodal position presented on the y-axis with 1 being the node closest to the shoot base. The dotted lines mark the nodal positions that were sampled in V1 and T2 according to Table 1.

The total plant height increased for C17 from 39.9 ± 1.5 cm at V1 to 53.1 ± 1.5 cm at T2 and 60.9 ± 2.1 cm at G3. Afterwards total length remained stable (61.0 ± 2.2 cm at G4 and 61.5 ± 2.4 cm at G5). C17 was characterised by vegetative growth at V1 and floral transition at T2. Thereafter, the shoot apex no longer initiated axillary buds and total number of nodal positions stayed at 47 from G3 to G5 (Fig 3). The vegetative growth was marked by apical dominance and short inhibited axillary bud outgrowth near the shoot apex in V1 (positions 37 to 31) and T2 (positions 45 to 38). Once generative growth started at G3 to G5, apical dominance was released and a strong outgrowth of the axillary shoots under the apex (positions 47 to 42) was observed. At V1 and T2, the axillary buds in the middle positions were under less influence by the apex and had larger axillary buds (positions 30 to 16 for V1 and 37 to 31 for T2) with even bud outgrowth in T2 at positions 30 to 16. The positions 15 to 1 under the middle showed strongly inhibited bud outgrowth as they were influenced by correlative inhibition (actively growing buds inhibiting the outgrowth of underlying axillary buds). From G3 to G5, shoot outgrowth decreased from position 41 to 38 and was completely inhibited for the lower positions (37 to 1).

**Fig 4:**
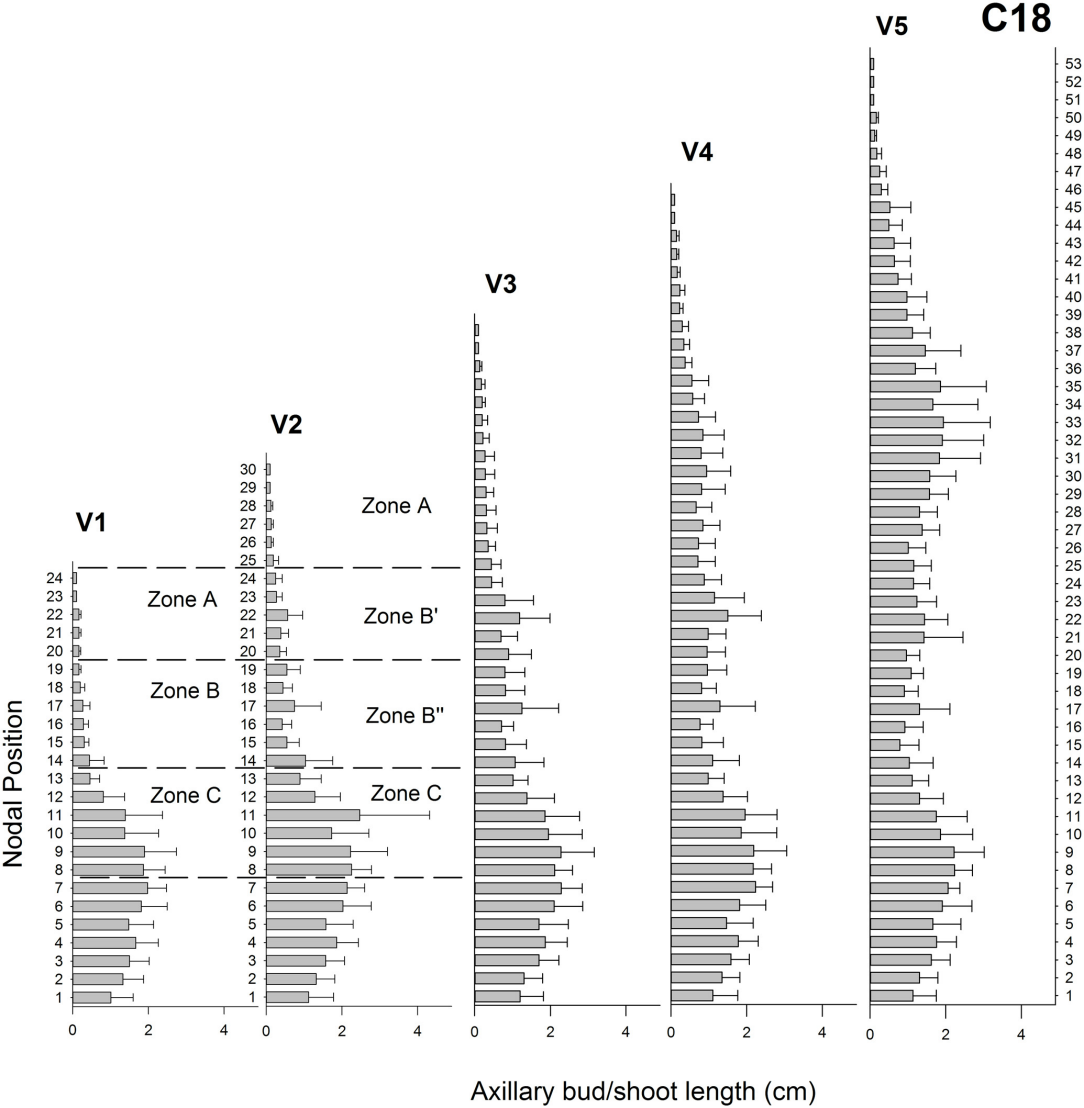
Axillary bud lengths for chrysanthemum C18 in V1 to V5. Mean bud/shoot length (±SE; n = 10) is presented on the x-axis for every nodal position presented on the y-axis with 1 being the node closest to the shoot base. The dotted lines mark the nodal positions that were sampled in V1 and V2 according to Table 1.

Plant heights for C18 during vegetative growth from V1 to V5 were 41.8 ± 3.1 cm, 58.3 ± 3.4 cm, 72.4 ± 3.3 cm, 83.6 ± 4.4 cm and 93.5 ± 8.1 cm during V1 to V5, respectively. The shoot apex did not undergo floral transition and continuously initiated new leaves. At V5, the number of nodal positions reached a maximum of 53. A strong apical dominance was manifested during the whole vegetative growth of C18 (Fig 4). This was revealed by the short axillary buds under the apex and an average axillary bud outgrowth of 1.27 ± 0.03 cm at V5.

The measurements of axillary buds were used to show the inhibition or activation of the nodal positions. Based on these measurements the nodal positions along the stem were subdivided in Zone A, Zone B and Zone C according to bud outgrowth activity and influences from apical dominance as described in Table 1. In C17 the nodal positions 30–1 at T2 (Fig 2) were not included because bud outgrowth had already occurred there and only samples of axillary buds were taken. These subdivisions (also indicated in Figs 2–4) were subsequently used in the hormone measurements and gene expression analysis.

### Levels of IAA and CK Change Differently at Floral Transition in C17 and during Vegetative Growth in C18

Raw data of CK and IAA measurements are provided in the supplemental information S7 Table. One of the three biological replicates was excluded for IAA (in C17 T2 Bud and Stem Zone A) and for CK (in C17 T2 Stem Zone B and in C18 V2 Bud Zone A); for these samples only 2 biological replicates were averaged.

**Fig 5:**
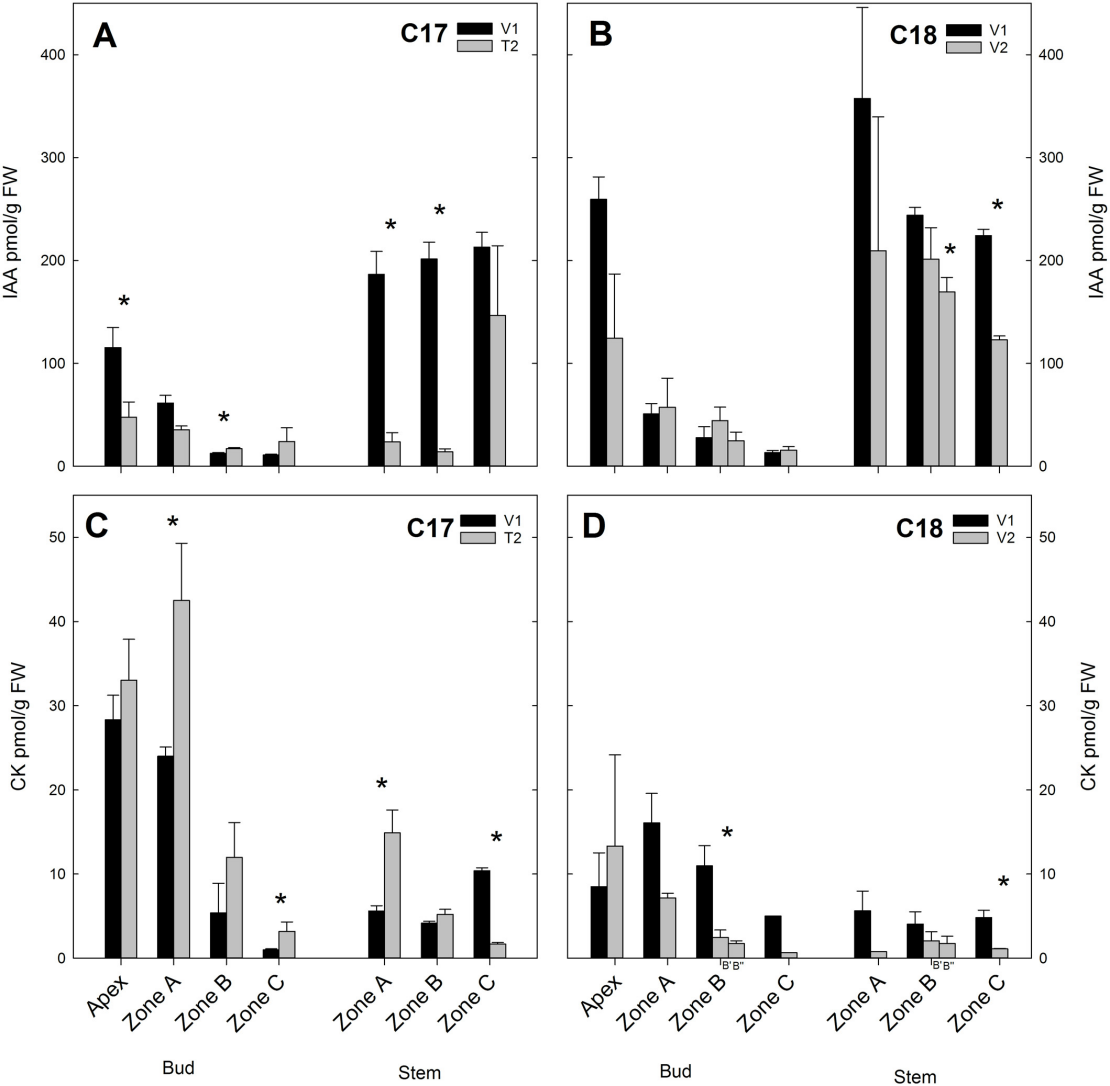
Hormone concentrations measured in two Chrysanthemum genotypes C17 and C18 at time point V1 and T2/ V2. IAA and cytokinin (CK) content of the shoot apex and the axillary buds or stem in different zones are presented. Data are means ± SE (n = 3). * indicates significance at the 0.05 level between V1 and T2/V2.

Overall, IAA levels were higher in C18 than in C17, especially in apex and stem (Fig 5A and 5B). For C17 at V1, IAA content in the shoot apex was significantly higher than in the axillary buds and significantly lower compared to the stem (Table 2). At T2 the IAA content in the apex had decreased and was similar to IAA levels in axillary buds and stem, except for zone B. Between different zones of axillary buds, IAA levels were higher in zone A at V1 but not different at T2. Between stem zones, IAA levels were similar at V1 but higher in zone C at T2 S8 Table.

**Table 2:** Kruskal-Wallis tests comparing the hormone levels between the shoot apex and the different zones of the axillary buds and stems. Data are fold changes (Zone X/Apex) and the significant difference between means by Kruskal-Wallis test is indicated by * (p-value<0.05).

C17		Bud			Stem				
		Apex-A	Apex-B	Apex-C	Apex-A	Apex-B	Apex-C		
V1	IAA	-1.9*	-9.2*	-10.4*	1.6*	1.8*	1.8*		
	CK	-1.2	-5.3*	28.6*	-5.1*	-6.8*	2.7*		
T2	IAA	-1.3	-2.8*	-2.0	-2.01	-3.4*	3.07		
	CK	1.3	-2.8*	10.4*	-2.2*	-6.4*	19.9*		
C18		Bud				Stem			
		Apex-A	Apex-B’	Apex-B”	Apex-C	Apex-A	Apex-B’	Apex-B”	Apex-C
V1	IAA	-5.1*	-9.3*		-19.4*	1.38	-1.06		-1.16
	CK	1.89	1.29		1.70	-1.51	-2.10		1.76
V2	IAA	-2.17	-2.80	-5.04	-3.64	1.68	1.62	1.36	-1.01
	CK	-1.87	-5.43	-7.65	-10.7*	-16.83	-6.45	-7.62	-12.1*

**Fig 6:**
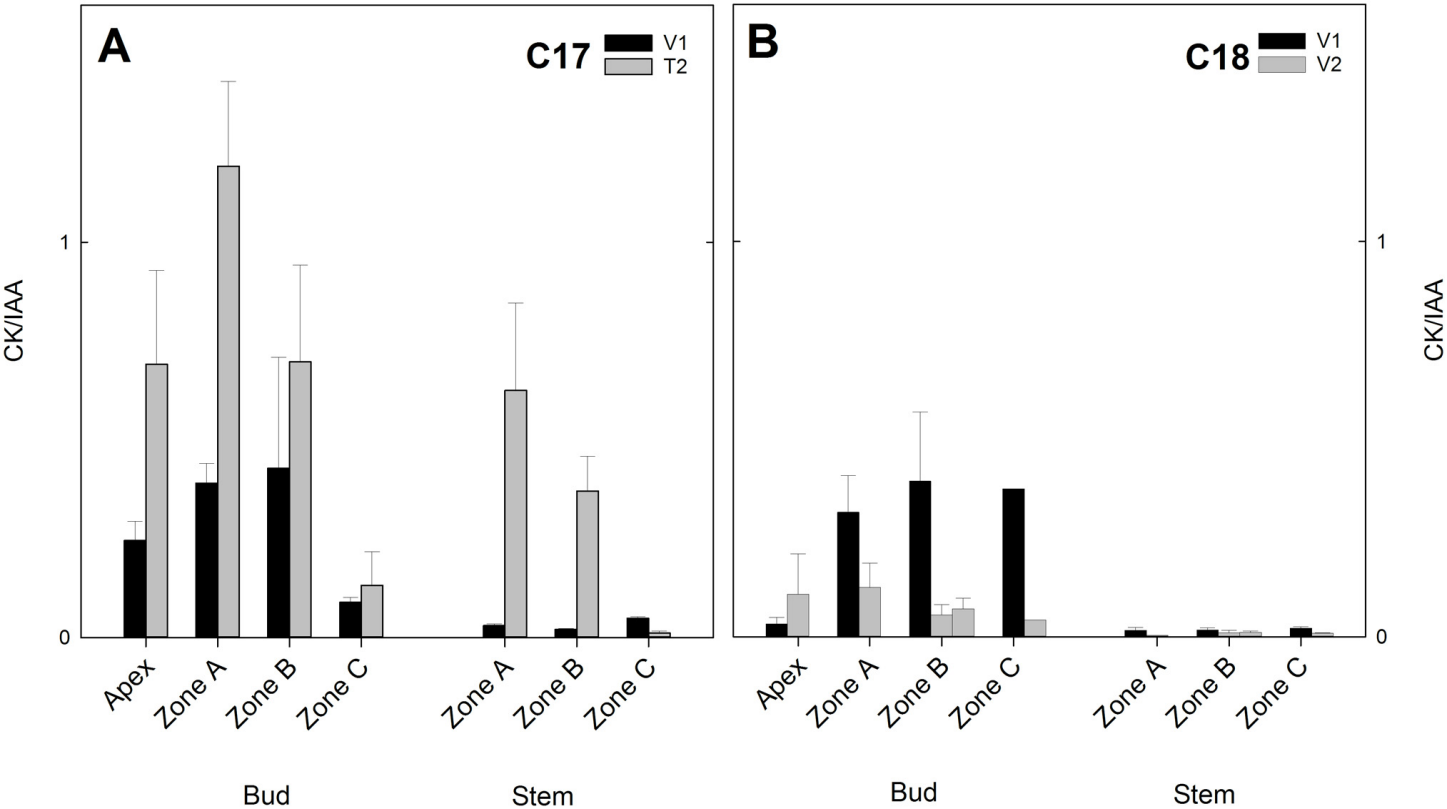
CK/IAA ratio for Chrysanthemum genotypes C17 and C18 at two time points V1 and T2/V2. Total cytokinin/IAA of the shoot apex and the axillary buds or stem in different zones are presented. Data are mean cytokinin levels (n = 3) divided by mean auxin levels (n = 3) + calculated SE.

Changes in CK/IAA ratio reflected the altered hormone levels in C17 at floral transition and in C18 during vegetative growth. In C17 the CK/IAA ratio increased from V1 to T2, most notably in the apex and in zone A and B of both buds and stems (Fig 6A). The CK/IAA ratio in C18 decreased from V1 to V2 in the axillary buds and in the apex and stem (Fig 6B).

At the transition to generative growth in C17, from V1 to T2, a decrease in IAA content was observed in the apex and axillary buds of zone A, while a slight increase was seen in zone B and C. In the stem a significant decrease in auxin level occurred in zone A and B (Fig 5A).

IAA content in the shoot apex of C18 was significantly higher than in the axillary buds but did not differ from the IAA content in the stem at V1 (Table 2). At V2, the IAA content in the shoot apex did not differ significantly from the axillary buds or stem (Table 2). Between zones of axillary buds, no differences were seen at V1 or V2. Between stem zones no differences were seen at V1 but at V2 zone B’ and B” differed significantly from zone C S8 Table.

In C18 there was no significant difference in IAA levels between V1 and V2 in the apex and axillary buds. In the stem a significant decrease was observed in zone B” and zone C but the absolute auxin level remained high compared to the stem zone A and B of C17 (Fig 5B).

Cytokinin (CK) content was generally higher in C17 when compared to C18, especially in apex and axillary buds (Fig 5C and 5D). CK content in the shoot apex was significantly higher than in the axillary buds (except for zone A) and the stem in C17 at V1 (Table 2). At T2, CK content in the shoot apex remained significantly different from the axillary buds (except zone A) and stem (except zone B) (Table 2). Between different zones of axillary buds, CK levels differed significantly and were highest in zone A and lowest in zone C at V1 and V2. CK content between all stem zones differed at V1 but at T2 only zone A and zone C were significantly different S8 Table.

At floral transition from V1 to T2 in C17, the CK content increased significantly in the axillary buds of zone A and C and in the stem of zone A (Fig 5C).

For C18 the CK content in the apex showed no significant differences with axillary buds and stem at V1 or V2 (except with zone C at V2). Between zones of axillary buds or stem no differences were seen at V1 or V2 S8 Table.

In C18, CK levels had a decreased trend from V1 to V2 throughout the axillary buds and stem with significant changes in bud Zone B’ and stem zone C.

### Floral Transition in C17 Shows a Different Gene Expression Profile Compared to Vegetative Growth in C18

#### Isolation of branching genes

In total 15 fragments of orthologous candidate branching genes, *CmDRM1* (Acc. Nr. KU528658), *CmSTM* (Acc. Nr. KU528663), *CmMAX1* (Acc. Nr. KT124645), *CmMAX3* (Acc. Nr. KU528660), *CmRR1* (Acc. Nr. KU528651), *CmHK3a* (Acc. Nr. KU528656), *CmHK3b* (Acc. Nr. KU528657), *CmTIR3* (Acc. Nr. KU528665), *CmPIN1* (Acc. Nr. KU528662), *CmAXR1* (Acc. Nr. KU528652), *CmTIR1* (Acc. Nr. KU528664), *CmAXR6* (Acc. Nr. KU528654), *CmIAA12* (Acc. Nr. KU528659), *CmAXR2* (Acc. Nr. KU528653) and *CmIAA16* (Acc. Nr. KU528655), and 11 reference genes *CmUBC* (Acc. Nr. KX586319), *CmUBQ10* (Acc. Nr. KX586320), *CmEF1α* (Acc. Nr. KX586321), *CmACT2* (Acc. Nr. KX586322), *CmATUB* (Acc. Nr. KX586323), *CmCACS* (Acc. Nr. KX586324), *CmEXP5* (Acc. Nr. KX586325), *CmEXP6* (Acc. Nr. KX586326), *CmPGK* (Acc. Nr. KX586327), *CmPSAA* (Acc. Nr. KX586328) and *CmHH3* (Acc. Nr. KX586329), were isolated in chrysanthemum and their sequences were deposited in the NCBI nucleotide database. The isolated branching gene sequences were verified by BLASTX against the NCBI database; the closest hits all corresponded to the gene of interest (Table 3). Additionally, the functional domains were indicated on the isolated sequences, corresponding to the same functional domains in the Arabidopsis sequences S2 Fig. For *CmHK3*, two different fragments *CmHK3a* and *CmHK3b* were isolated which shared a 96.99% identity in their nucleic acid sequence (Clustal Omega [69]).

**Table 3:** BlastX search of the isolated cDNA sequences with E-value, % identity and accession numbers.

	Gene	Description	E-value	%Ident	Accession N°
*Bud development*	***CmDRM1***	auxin-repressed 12.5 kDa protein [*Jatropha curcas*]	1,00E-23	64	NP_001295697.1
		auxin-repressed protein [*Nicotiana tabacum*]	6,00E-22	65	AAS76635.1
		dormancy-associated protein-like 1 [*Arabidopsis thaliana*]	5,00E-21	56	NP_564305.1
	***CmSTM***	knotted-1-like protein 1 [*Helianthus annuus*]	5,00E-37	98	AAM28231.1
		shootmeristemless-like [*Petunia x hybrida*]	1,00E-35	98	AAM47027.1
		shoot meristemless [*Arabidopsis thaliana*]	4,00E-34	97	ABR09190.1
*Strigolactone*	***CmMAX1***	MAX1 [*Petunia x hybrida*]	2,00E-59	85	AEB97383.1
		PREDICTED: cytochrome P450 711A1 [*Solanum lycopersicum*]	3,00E-58	85	XP_004245085.1
		cytochrome P450 monooxygenase [*Arabidopsis thaliana*]	1,00E-55	79	ABR08959.1
	***CmMAX3***	more axillary branching 3 [*Artemisia annua*]	9,00E-51	95	ADB64459.1
		carotenoid cleavage dioxygenase 7 [*Actinidia chinensis*]	5,00E-40	79	ADP37985.1
		carotenoid cleavage dioxygenase 7 [*Arabidopsis thaliana*]	7,00E-35	71	NP_182026.4
*Cytokinin*	***CmRR1***	PREDICTED: ARR1-like isoform X1 [*Pyrus x bretschneideri*]	2,00E-21	42	XP_009369970.1
		PREDICTED: ARR1-like isoform X1 [*Malus domestica*]	2,00E-19	42	XP_008340753.1
		PREDICTED: ARR1-like isoform X1 [*Fragaria vesca*]	9,00E-17	41	XP_004295112.1
	***CmHK3a***	PREDICTED: histidine kinase 3 isoform X1 [*Jatropha curcas*]	2,00E-83	80	XP_012085699.1
		Histidine kinase 3 [*Glycine soja*]	1,00E-82	79	KHN12383.1
		PREDICTED: histidine kinase 3-like [*Solanum tuberosum*]	3,00E-79	78	XP_006352176.1
	***CmHK3b***	PREDICTED: histidine kinase 3 [*Glycine max*]	1,00E-61	80	XP_003524900.1
		PREDICTED: histidine kinase 3 isoform X1 [*Jatropha curcas*]	2,00E-61	81	XP_012085699.1
		histidine kinase 3 [*Arabidopsis thaliana*]	1,00E-59	79	NP_564276.1
*Auxin*: *transport*	***CmPIN1***	auxin efflux carrier component 1-like [*Cucumis sativus*]	2,00E-44	91	NP_001275530.1
		auxin transporter PIN1 [*Triticum aestivum*]	3,00E-43	88	AAS19858.1
		auxin efflux carrier component 1 [*Agave tequilana*]	3,00E-43	88	AJI44018.1
	***CmTIR3***	PREDICTED: auxin transport protein BIG [*Vitis vinifera*]	2,00E-45	67	XP_010660565.1
		Auxin transport protein BIG [*Glycine soja*]	5,00E-39	59	KHN45099.1
		auxin transport protein BIG [*Arabidopsis thaliana*]	6,00E-32	55	NP_186875.2
*Auxin*: *signalling*	***CmTIR1***	transport inhibitor response 1 [*Cynara cardunculus* var. scolymus]	2,00E-86	92	AFM95208.1
		Protein TRANSPORT INHIBITOR RESPONSE 1 [*Glycine soja*]	1,00E-80	86	KHN28237.1
		transport inhibitor response 1 [*Arabidopsis thaliana*]	8,00E-75	85	ADL70210.1
	***CmAXR1***	NEDD8-activating enzyme E1 regulatory subunit [*Vitis vinifera*]	1,00E-103	87	XP_002267415.1
		NEDD8-activating enzyme E1 regulatory subunit [*Ricinus communis*]	6,00E-100	83	XP_002524186.1
		RUB-activating enzyme E1 [*Arabidopsis thaliana*]	2,00E-89	73	NP_973761.1
	***CmAXR6***	cullin 1 [*Vitis vinifera*]	2,00E-101	95	ACA30309.1
		Cullin-1, putative [*Ricinus communis*]	2,00E-94	95	XP_002516899.1
		cullin1 [*Catharanthus roseus*]	9,00E-94	94	ALI87038.1
*Auxin*: *response*, *Aux/IAA*	***CmAXR2***	Auxin-responsive protein IAA7 [*Morus notabilis*]	2,00E-79	84	XP_010087762.1
		Auxin-responsive protein IAA7, putative [*Ricinus communis*]	2,00E-79	83	XP_002517023.1
		PREDICTED: auxin-responsive protein IAA7 [*Brassica napus*]	2,00E-76	81	XP_013749219.1
	***CmIAA16***	auxin-responsive AUX/IAA family protein [*Medicago truncatula*]	6,00E-53	94	XP_013449825.1
		Auxin-responsive protein IAA16, putative [*Ricinus communis*]	8,00E-52	92	XP_002531288.1
		PREDICTED: auxin-responsive protein IAA16 [*Jatropha curcas*]	7,00E-51	90	XP_012089266.1
	***CmIAA12***	bodenlos family protein [*Populus trichocarpa*]	5,00E-44	70	XP_002311698.2
		PREDICTED: auxin-responsive protein IAA12-like [*Nelumbo nucifera*]	9,00E-43	69	XP_010262457.1
		PREDICTED: auxin-responsive protein IAA12 [*Malus domestica*]	1,00E-39	66	XP_008340670.1

### Gene Expression Analysis

Rt-qPCR was done using the 15 isolated genes and 4 genes previously isolated in chrysanthemum: *CmBRC1* [53], *CmLSL* [54], *CmIPT3* (Acc. Nr. JQ711176.1) and *CmMAX2* [58]. These genes represent key signalling functions in bud development and the pathways of auxin, cytokinin and strigolactones (Fig 1B). In the expression analysis of *CmMAX3 t*here was amplification in the no-RT for the majority of the samples and the ΔCp cut-off- value of 5 was not met. Therefore *CmMAX3* expression was excluded from the analysis. Similarly, one of the three biological replicates was excluded for *CmAXR1* (in C17 V1 Bud Zone C), *CmHK3a* (in C17 V1 Bud Zone A), *CmTIR3* (in C17 V1 Bud zone A and in Bud Zone C V1 and T2), *CmAXR2* (in C18 V2 Bud zone B”) and *CmIPT3* (in C18 V2 Bud Zone A); for these samples only 2 biological replicates were averaged.

**Fig 7:**
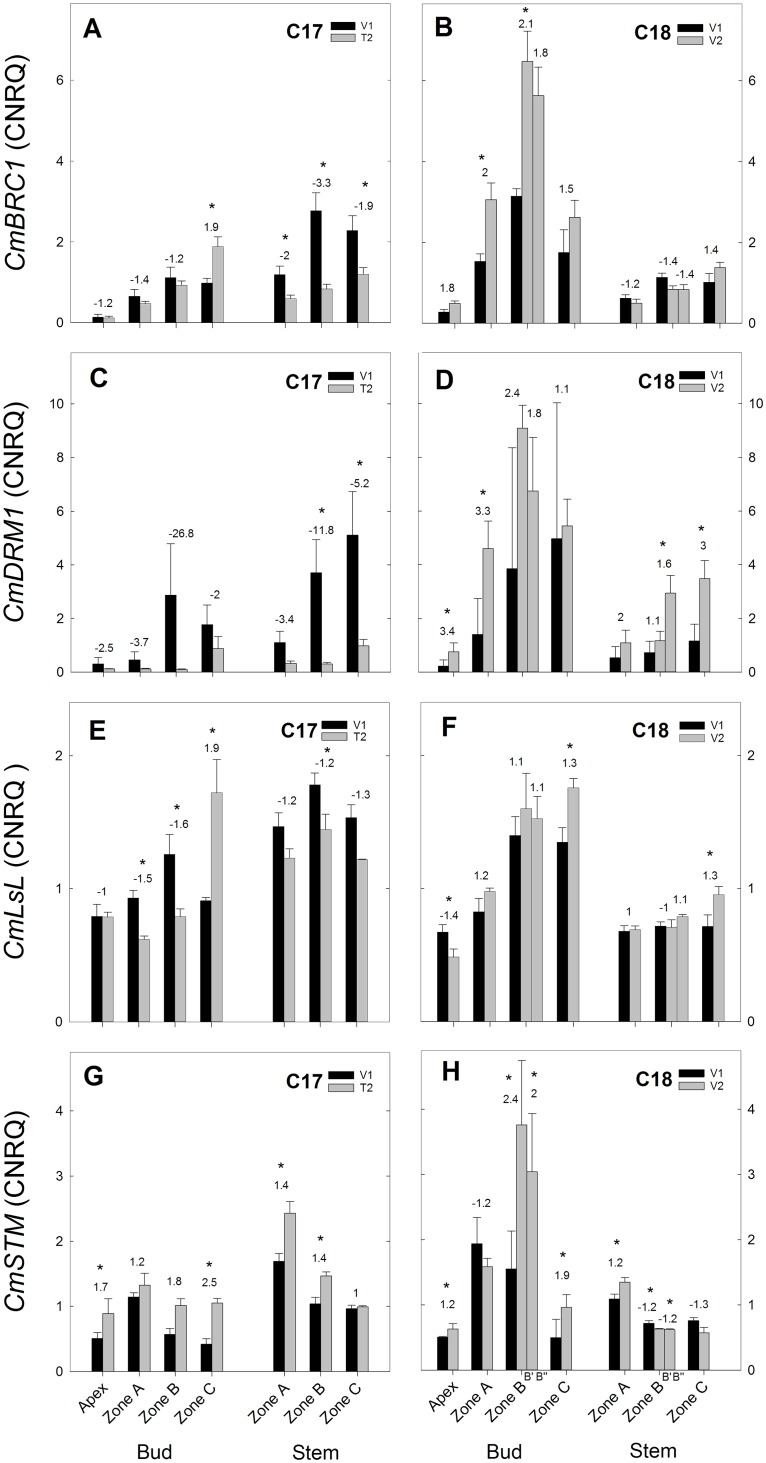
RT-qPCR gene expression analysis of bud development genes for Chrysanthemum genotypes C17 and C18 at time points V1 and T2/V2. CNRQ for bud development related genes *CmBRC1*, *CmDRM1*, *CmLsL* and *CmSTM* in the shoot apex, axillary buds and stem samples. Data are means ± SE (n = 3) of non log-transformed CNRQ. Fold changes between V1 and T2/V2 are indicated above the grey bars with * indicating significance of the Kruskal Wallis test (p<0.05).

**Fig 8:**
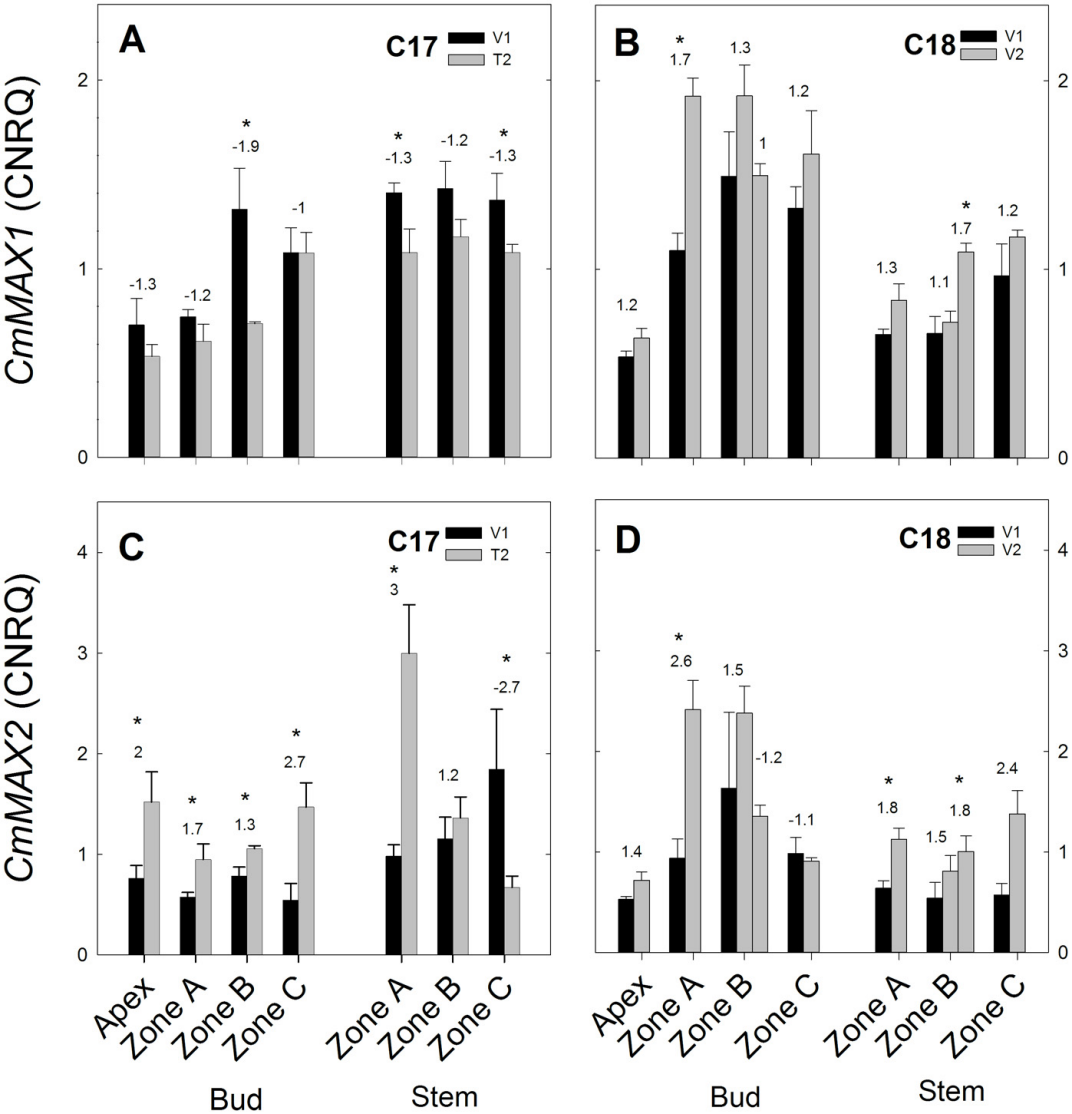
RT-qPCR gene expression analysis of strigolactone genes for Chrysanthemum genotypes C17 and C18 at time points V1 and T2/V2. CNRQ for strigolactone genes CmMAX1 and CmMAX2 in the shoot apex, axillary buds and stem samples. Data are means ± SE (n = 3) of non log-transformed CNRQ. Fold changes between V1 and T2/V2 are indicated above the grey bars with * indicating significance of the Kruskal Wallis test (p<0.05).

**Fig 9:**
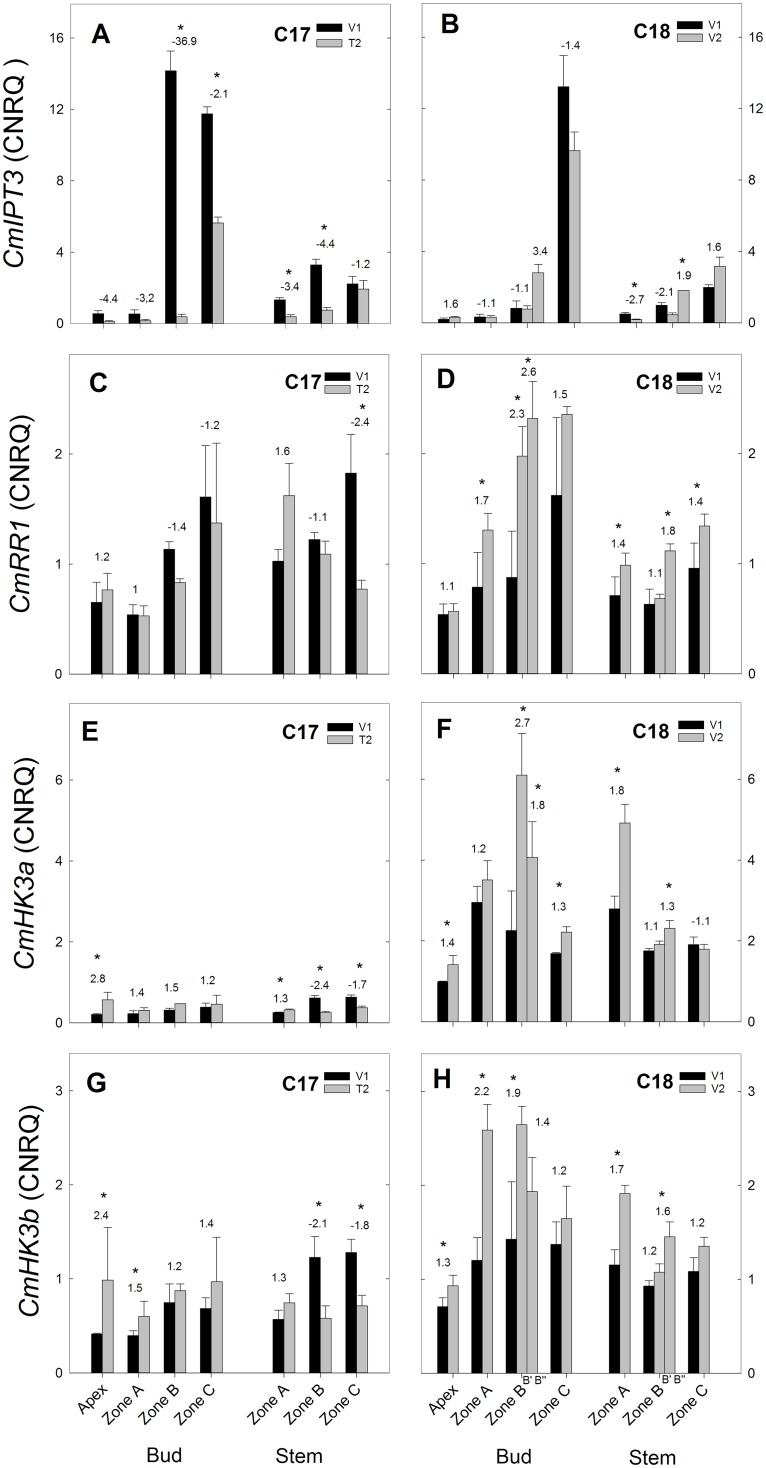
RT-qPCR gene expression analysis of cytokinin genes for Chrysanthemum genotypes C17 and C18 at time points V1 and T2/V2. CNRQ for cytokinin pathway genes CmIPT3, CmRR1, CmHK3a and CmHK3b in the shoot apex, axillary buds and stem samples. Data are means ± SE (n = 3) of non log-transformed CNRQ. Fold changes between V1 and T2/V2 are indicated above the grey bars with * indicating significance of the Kruskal Wallis test (p<0.05).

**Fig 10:**
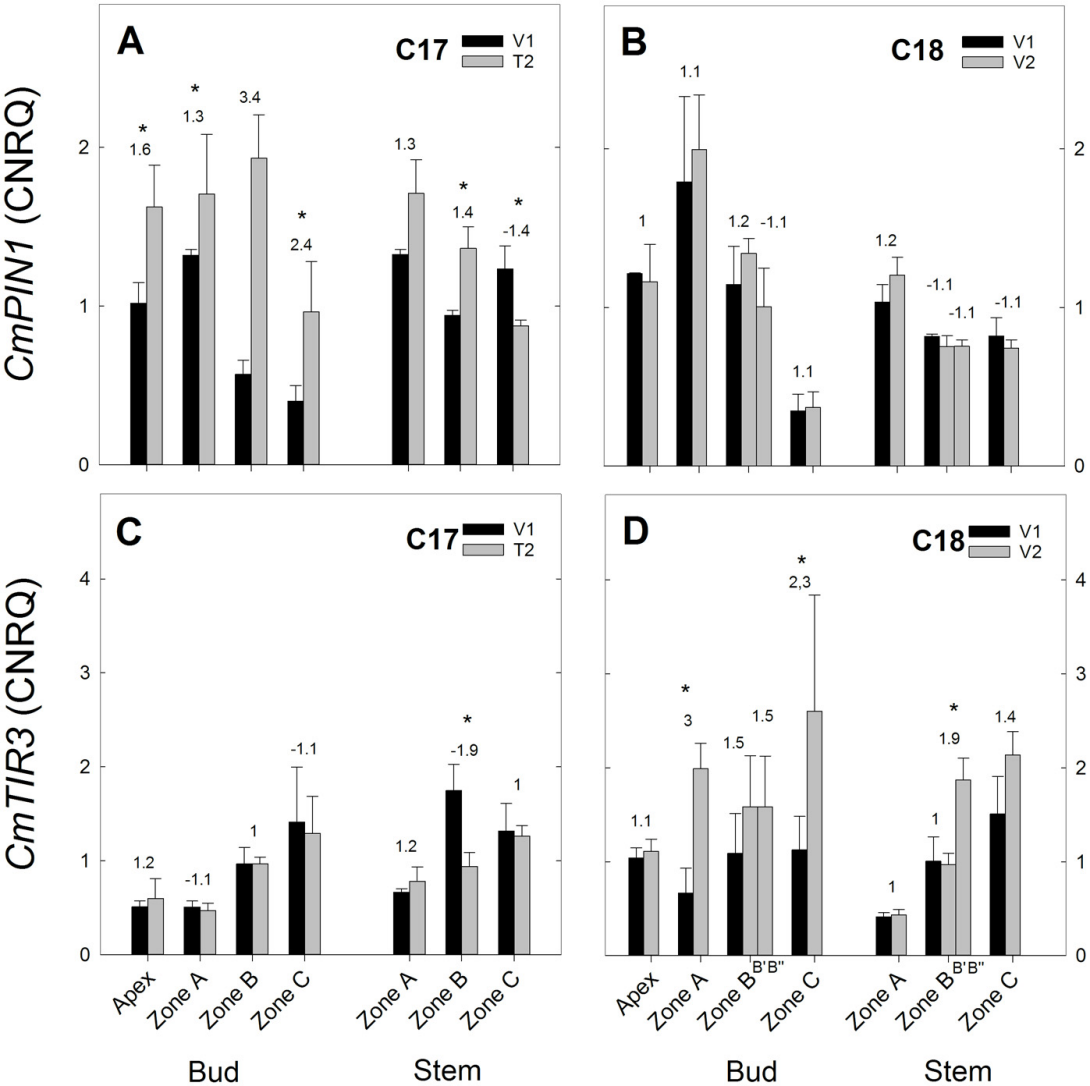
RT-qPCR gene expression analysis of auxin transport genes for Chrysanthemum genotypes C17 and C18 at time points V1 and T2/V2. CNRQ for auxin transport genes *CmPIN1* and *CmTIR3* in the shoot apex, axillary buds and stem samples. Data are means ± SE (n = 3) of non log-transformed CNRQ. Fold changes between V1 and T2/V2 are indicated above the grey bars with * indicating significance of the Kruskal Wallis test (p<0.05).

**Fig 11:**
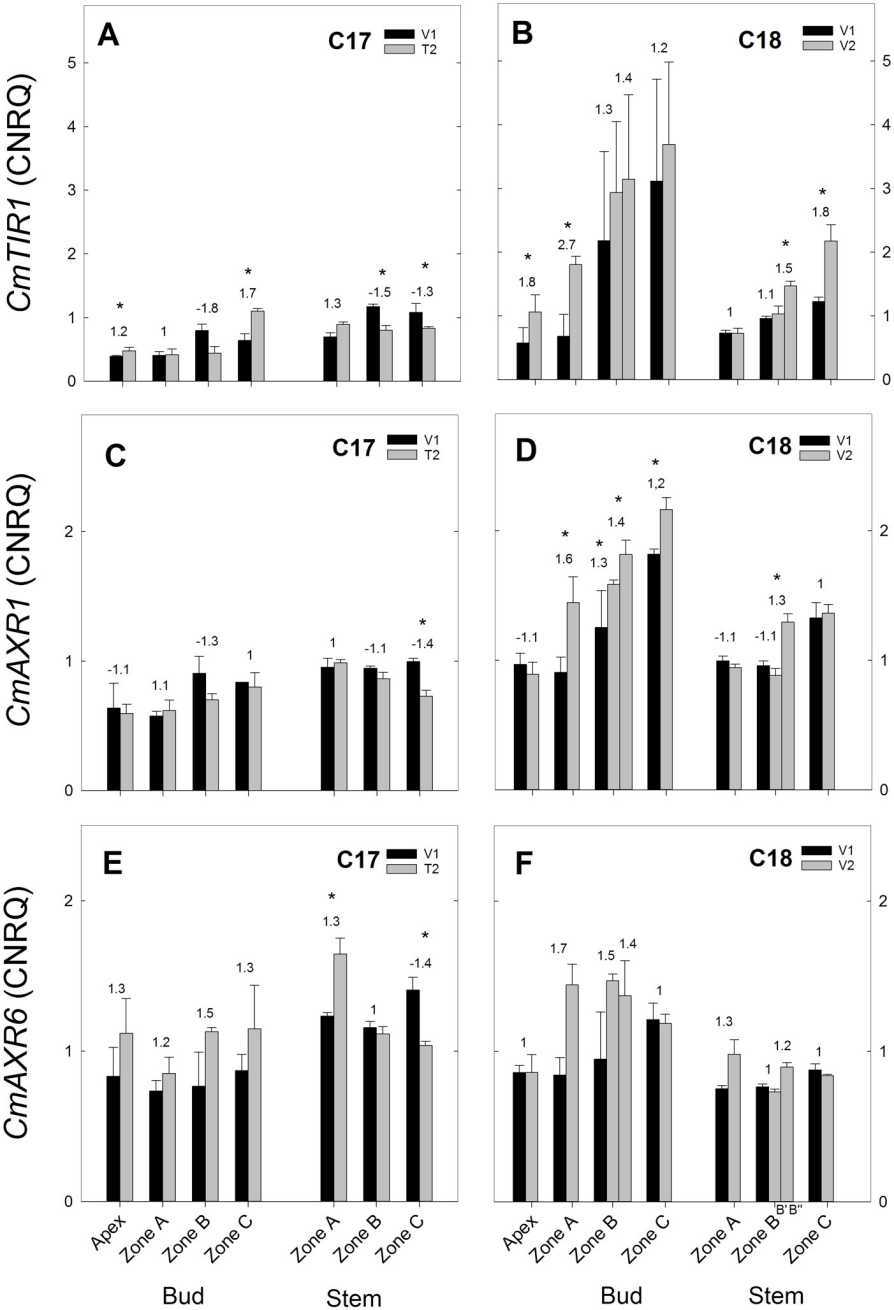
RT-qPCR gene expression analysis of auxin signalling genes for Chrysanthemum genotypes C17 and C18 at time points V1 and T2/V2. CNRQ for auxin perception genes *CmTIR3*, *AXR1 and AXR6* in the shoot apex, axillary buds and stem samples. Data are means ± SE (n = 3) of non log-transformed CNRQ. Fold changes between V1 and T2/V2 are indicated above the grey bars with * indicating significance of the Kruskal Wallis test (p<0.05).

**Fig 12:**
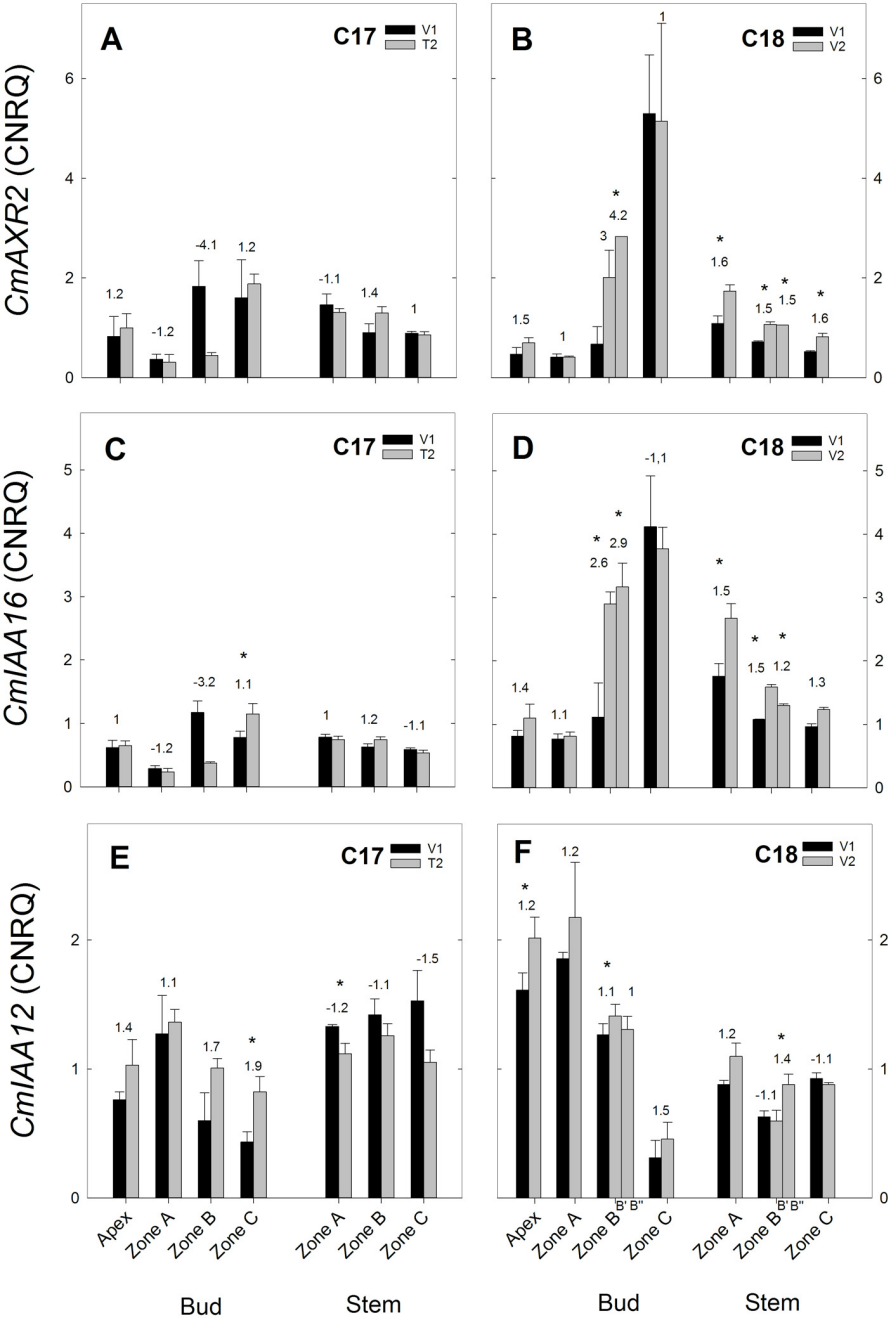
RT-qPCR gene expression analysis of auxin response genes for Chrysanthemum genotypes C17 and C18 at time points V1 and T2/V2. CNRQ for auxin response genes *CmAXR2*, *CmIAA16 and*, *CmIAA12* in the shoot apex, axillary buds and stem samples. Data are means ± SE (n = 3) of non log-transformed CNRQ. Fold changes between V1 and T2/V2 are indicated above the grey bars with * indicating significance of the Kruskal Wallis test (p<0.05).

The expression of these genes is presented separately for C17 and C18 with significant differences between V1 and T2/V2 indicated on the figures (Figs 7–12). Raw CNRQ expression values are available in S10 Table. Additional comparisons were made between the apex and zones of axillary buds and between the zones of the stem at each time point and between time points for C17 (S11–S13 Tables) and C18 (S14–S16 Tables).

#### Expression of bud development genes

The expression of the bud development related genes *CmBRC1* and *CmDRM1* showed a different pattern in C17 compared to C18. A decreased trend from V1 to T2 was seen in the expression of *CmBRC1* (Fig 7A) and *CmDRM1* (Fig 7C), while an increased trend was observed in C18 (Fig 7B and 7D). This decreased expression in C17 was most pronounced in the stem with significant changes in all zones for *CmBRC1* and in zone B and C for *CmDRM1*. In C18 increased expression of *CmBRC1* was significant in axillary bud zone A and B’, while *CmDRM1* expression increased significantly in apex, axillary bud zone A and stem zone B” and C.

*CmLsL* expression decreased in C17 from V1 to T2 in the axillary buds of zone A, B and C and in the stem zone B (Fig 7E). In C18 *CmLsL* expression increased in shoot apex and in the axillary buds of zone C from V1 to V2 (Fig 7F). *CmSTM* expression increased both in C17 (from V1 to T2 in apex, bud zone C and stem zone A,B) and in C18 (from V1 to V2 in apex, bud zone B, C and stem zone A).

#### Expression of strigolactone genes

The strigolactone biosynthetic gene *CmMAX1* was expressed differently in C17 and C18. A decreasing trend from V1 to T2 in C17 (Fig 8A and 8E) and an increased expression from V1 to V2 in C18 (Fig 8B and 8F) was observed. Decreased expression in C17 was significant in bud zone B and stem zone A and C for *CmMAX1*. Increased expression in C18 was significant in bud zone A and stem zone B” for *CmMAX1*. The expression of the strigolactone signalling gene *CmMAX2* increased in C17 from V1 to T2 and in C18 from V1 to V2.

#### Expression of cytokinin genes

Expression of cytokinin biosynthesis gene *CmIPT3* in C17 showed an overall decrease from V1 to T2 (Fig 8A). In C18 no significant difference in expression was observed between V1 and V2, except in zone A and B of the stem (Fig 8B). Cytokinin receptor genes *CmHK3a* and *CmHK3b* (2 fragments of the *CmHK3* gene) showed similar expression patterns in C17 and C18 but with higher expression of *CmHK3a* in C18 compared to *CmHK3b*. In C17 expression increased in apex and decreased in the stem (Fig 8E and 8G). In C18, an increased trend was seen from V1 to V2 in apex, bud and stem (Fig 8F and 8H). The cytokinin response gene *CmRR1* showed little change in expression from V1 to V2 in C17 except for a significant decrease in stem zone C (Fig 9C). In C18 *CmRR1* expression showed an increasing trend from V1 to V2 with significant changes in bud zone A, B and stem zone A, B” and C (Fig 9D).

#### Expression of auxin genes

The auxin transport gene *CmPIN1* showed an increased expression in C17 from V1 to T2, but a significant decrease was seen in stem zone C (Fig 10A). In C18 no significant changes in *CmPIN1* expression were observed between V1 and V2 (Fig 10B). *CmTIR3* expression showed little change in C17 from V1 to T2 with one significant decrease in stem zone B (Fig 10C). In C18 an increased trend was seen with significant changes in bud zone A, C and stem zone B” (Fig 10D).

The auxin perception genes *CmTIR1* and *CmAXR1* had a similar expression pattern in C17 and C18. Their expression in C17 from V1 to T2 remained low or largely unchanged (Fig 11A and 11C), while in C18, an increased trend was seen from V1 to V2 (Fig 11B and 11D). *CmAXR6* expression in C17 showed little change from V1 to T2 with a significant increase in stem zone A and a significant decrease in stem zone C (Fig 11E). In C18 no significant changes were seen from V1 to V2 (Fig 11F).

The auxin response genes *CmAXR2*, *CmIAA16 CmIAA12* showed a largely similar pattern of expression with little change in C17 and an increased trend in C18. This was most evident for *CmAXR2* and *CmIAA16*. *CmAXR2* expression showed no significant differences from V1 to T2 in C17 (Fig 12A) and in C18 *CmAXR2* expression increased significantly from V1 to V2 in bud zone B” and stem zone A, B, C (Fig 12B). *CmIAA16* expression showed little difference from V1 to T2 (Fig 12C), while in C18 an increased trend of *Cm IAA16* expression was seen from V1 to V2 with significant changes in bud zone B and stem zone A, B (Fig 12D). *CmIAA12* expression in C17 from V1 to T2 showed an increased trend in apex and buds and a decreased trend in the stem (Fig 12E). In C18 an increased trend from V1 to V2 was seen with significant changes in apex, bud zone B’ and stem zone B” (Fig 12F).

Additional hierarchical clustering S3 Fig. and canonical discriminant function analysis S4 Fig. further revealed some broader trends in the gene expression occurring in the genotypes C17 and C18. Hierarchical clustering showed a close linkage between auxin response genes *CmAXR2* and *CmIAA16* in both C17 and C18. In C18 *CmBRC1* was closely linked to *CmMAX1* and not far removed from *CmDRM1*. In C17, *CmDRM1* and *CmMAX1* were also found to be close to each other. This matches the trend of the general increased *CmAXR2* and *CmIAA16* expression in C18 versus the unaltered expression of these genes in C17. Also *CmMAX1*, *CmBRC1* and *CmDRM1* shared a similar trend of general decreased expression at T2 in C17 and increased expression at V2 in C18. The canonical discriminant function analysis showed a distinct clustering of apex samples and of the different zones in the axillary bud and stem samples in both C17 and C18 S4A and S4D Fig with stem samples clustered closely together. In C17 the floral transition at T2 leads to a clustering of Bud zone A and B, apart from the stem zones and bud zone C S4C Fig. This distinctive clustering between bud and stem zones is less pronounced in C17 at V1 S4B Fig or in C18 at V1 and V2 S4E and S4F Fig. This clustering reflects the zonation in gene expression of apex, axillary bud and stem samples with a clear difference between the two time points in C17, a difference that is less pronounced in C18.

## Discussion

The axillary shoot growth observed in both chrysanthemum genotypes C17 and C18 was determined by apical dominance during vegetative growth. This was shown in C17 by the inhibition of axillary buds immediately under the apex, the larger buds in the middle part and the inhibited lower buds at V1 (Fig 2). In C18 the stronger apical dominance was evidenced by the overall inhibited axillary buds in the vegetative growth (Fig 4). These branching forms matched with previous axillary bud/shoot length measurements in chrysanthemum cut flowers [53,70]. In C17 transition to the generative growth phase at T1 caused a release from apical dominance followed by the growth of axillary shoots directly under the apex. This concurs with previous measurements of subapical axillary bud outgrowth in chrysanthemum after release from apical dominance by decapitation and floral transition [53]. At the end of the experiment, both genotypes exhibited their typical growth habitus, a split type for C17 and a continuous vegetative growth under long day for C18.

The hypothesis of apical dominance where a high auxin production in the shoot apex and subsequent basipetal auxin transport inhibits the outgrowth of axillary buds is supported by various studies on vegetative axillary buds [70–72]. The reported IAA contents in the shoot apex, stem and axillary buds in this study were in agreement with this apical dominance mechanism (Fig 5). The higher IAA content measured in the shoot apex of C18 compared to C17 corresponded with the stronger apical dominance observed in C18. The floral transition initiated at T2 in C17 coincided with a decreased IAA content in the shoot apex and in the stem. In C18 no floral transition took place and correspondingly no drastic decrease of IAA content was observed from V1 to V2.

Cytokinins are known regulators of axillary bud outgrowth and cytokinin homeostasis is also influenced by auxin that can inhibit biosynthesis and promote degradation [27]. The increased CK levels observed in the axillary buds and stem in zone A of C17 at the moment of floral initiation (T2) reflect this axillary bud outgrowth promoting role. In vegetatively growing C18 plants, CK levels decreased in the axillary buds and stem from V1 to V2. Furthermore, lower CK levels were measured in the shoot apex, axillary buds and stem of C18, which exhibited a stronger apical dominance than C17 (Fig 5). Like these observations, previous reports have shown increased cytokinin levels in axillary buds at the release from apical dominance by apex defoliation in tobacco plants [73] and by decapitation in pea plants [14].

The CK/IAA ratio offers a good indicator for the state of apical dominance and axillary bud outgrowth as was shown previously by Emery et al. [72] in lupin. In that case inhibited axillary buds showed a decreased CK/IAA ratio, whereas activated axillary buds showed increasing CK/IAA ratios. An increased CK/IAA ratio was also described in the outgrowth of axillary buds of tobacco following release from apical dominance [73]. In chrysanthemum we observed a strong increase in CK/IAA ratio in C17 in the subapical axillary buds of zone A at the time of release from apical dominance by floral initiation. In C18, where floral transition is absent, there was a notable decrease of the CK/IAA ratio in the axillary buds (Fig 6).

In the subdivision of the different samples of axillary buds and stem into the zones A, B and C it could be remarked that in C17 the nodal positions at T2 do not match those in V1. These positions however where chosen to reflect a physiological change that was seen in the axillary bud outgrowth under the apex at the transition from vegetative growth to generative growth. Furthermore middle positions in T2 already showed bud outgrowth with side shoots. These nodes were omitted from the sampling to only include axillary buds. For this reason, the nodal positions in zone A at V1 are the same positions that become zone C in T2. The different physiological status that occurs at floral transition in T2 is however better reflected by the change from zone A V1 to zone A T2, with decreased auxin content and increased cytokinin content in axillary bud and stem. This change is not seen from zone A V1 to zone C T2 S8 Table. and this comparison would disregard information about the hormonal regulation that happens in the axillary buds under the apex that show a strong outgrowth after floral transition. This was also shown by the changes in gene expression that occurred from V1 to T2 in zone A, and not from zone A V1 to zone C T2 S13 Table., coinciding with the changing hormone levels.

In C17 *CmBRC1*, *CmDRM1* and *CmMAX1* expression showed a decreased trend from V1 to T2, while in C18 their expression had an increased trend or remained constant from V1 to V2 (Figs 7 and 8). This indicates that *CmBRC1 and CmDRM1* are downregulated at transition to generative growth and release from apical dominance in C17, while upregulation is seen in C18 during continuous vegetative growth. This corresponds with previous reports of high *BRC1* expression in chrysanthemum [53] and high *DRM1* expression in pea and Arabidopsis [30,31] in inhibited axillary buds and downregulation in activated buds.

It has to be noted here that in our results the most striking decrease in *CmBRC1* expression at floral transition happened in the stem samples, while in the axillary buds only a slight decrease was observed in the axillary bud samples of zone A and B with even an increase in zone C. This observation can be seen as somewhat contradictory with earlier reports of the role of *BRC1*. *BRC1* was found to be mainly expressed in dormant axillary buds in chrysanthemum and showed decreased transcript levels from 1 hour after release from apical dominance by decapitation [53]. Interestingly, in the same study, 48 hours after decapitation, the *BRC1* transcript levels had returned to the same levels as before the decapitation. This reported rapid downregulation of *BRC1* could be a possible explanation why we could not see a strong decrease in the axillary buds, assuming that *CmBRC1* levels had already reverted to the state before release of apical dominance by floral transition. On the other hand, it has been observed that in some cases the correlation between *BRC1* expression and axillary bud activity was weak. In rice for instance, expression of the *BRC1* orthologue *FC1*, is not reduced in dwarf10 (d10/max4) mutants that show increased shoot branching [74]. Also in maize the *TB1* expression did not decrease in ccd8 (max4) mutants with increased branching phenotype [75]. In our case it cannot be excluded that *CmBRC1* expression is correlated with axillary bud activity since a decrease in expression is still seen in the stem. To further study this relationship it would require samples at short intervals within 24 hours of the floral initiation to account for the rapid changes in *CmBRC1* expression.

The observed downregulation of *CmMAX1* in C17 suggests that local strigolactone biosynthesis in the stem and axillary buds decreases during transition to generative growth and release from apical dominance, preceding bud outgrowth in the axillary buds under the apex. This is the first description of *MAX1* in chrysanthemum. Given the role of strigolactones to inhibit axillary bud outgrowth [17] and also the regulation by *MAX1* in Arabidopsis [76] and in Petunia [46], our results suggest that the release from apical dominance at floral transition observed in chrysanthemum coincides with a reduced strigolactone inhibition as shown by a reduced *CmMAX1* expression. Expression levels of *MAX1* during floral transition have not been previously reported but in red clover *TpMAX1-1* expression as well as *TpBRC1* expression were downregulated in nodal stem segments 24 hours after bud outgrowth was induced by node excision [77].

The gene involved in auxin transport, *CmTIR3*, showed an increased expression from V1 to V2 in C18 while expression remained largely unaltered in C17 (Fig 10C and 10D). This observation corresponds well with the reported increased branching phenotype of Arabidopsis tir3 mutants at floral transition [12]. For *CmPIN1* however, a general increase in expression was seen in C17 while the *CmPIN1* expression in C18 remained constant (Fig 10A and 10B). It would indicate an increased auxin transport in C17 even though decreased auxin levels were observed here. This observation seems contradictory to what is known about auxin canalisation, where auxin promotes the transcription of *PIN1* through a positive feedback loop [78,79]. Furthermore it has been shown in Arabidopsis leaves that *PIN1* expression was decreased in *Tir3* mutant background, [80], so that a similar expression pattern of *CmPIN1 to CmTIR3* could be expected.

The expression of auxin signalling genes *CmTIR1* and *CmAXR1* and showed an increase in C18 from V1 to V2 while in C17 this was not observed. The general expression of these genes was also higher in C18 compared to C17 (Fig 11). For these genes however, previous reports have shown that auxin treatments did not increase their expression [38,81,82], making it less likely that the increase seen in C18 reflects a response to increased auxin levels.

*CmAXR2* and *CmIAA16* expression had an increased trend mainly in the stem of C18 (Fig 12). These Aux/IAA proteins function as repressors of auxin response but their transcription is induced by auxin [44,45]. Therefore the upregulation could be sign of a feedback to the auxin levels that remained high in C18 compared to C17. This pattern was less pronounced in the expression of *CmIAA12*.

The cytokinin genes showed a somewhat counterintuitive gene expression (Fig 9). For *CmIPT3* the decreased expression as seen in C17 stem and axillary buds from V1 to T2 should indicate decreased cytokinin biosynthesis. This is opposite from what would be expected by looking at the cytokinin levels that are increased in the top axillary buds and stem from week1 to week2. However in chrysanthemum *CmIPT3* has been previously reported to be upregulated in nodes after decapitation [53]. In Arabidopsis, *IPT3* is mainly expressed in the phloem and pericycle cells [83] and is upregulated by high nitrate [84]. Other cytokinin biosynthetic genes that are more important than *IPT3* might be involved in the cytokinin biosynthesis at the nodal positions. Possible candidates are *IPT1* and *IPT2* that are associated with raised cytokinin levels and biosynthesis in pea shoots preceding bud outgrowth after decapitation [14]. The cytokinin response genes *CmRR1*, *CmHK3a* and *CmHK3b* showed a general increased expression in C18 and some significant decreases in expression in C17. Like *CmIPT3* these results are not what would be expected from the observed cytokinin levels that increased in C17 and decreased in C18. Although it has been reported in Arabidopsis that several cytokinin response genes were downregulated in treatments with exogenous cytokinin [85]. A possible explanation could be that this cytokinin response can proceed without new protein synthesis. *CmHK3a* and *CmHK3b* represent 2 fragments that were isolated from the *CmHK3* gene and they share a 97% identity. The expression pattern of both fragments was similar along the apex, axillary buds and stem of genotypes C17 and C18 but in C18 the expression levels of *CmHK3a* were generally higher than *CmHK3b*.This suggests that *CmHK3a* has a different expression from *CmHK3b* and that these fragments could represent different forms of the CmHK3 gene such as is the case with the alternatively spliced *ZmHK3a* and *ZmHK3b* in maize [86] or the homologous *LjHK3a* and *LjHK3b* loci in lotus [87].

*CmLsL* was previously shown to be involved with shoot branching in chrysanthemum [55]. Overexpression resulted in increased branching and *LsL* expression was negatively correlated with IAA content in the shoot tip. From this we would expect to see an increase in *CmLsL* expression in C17 and a decrease in C18. In our experiments however we observed a general decrease in *LsL* expression in C17 which was not seen in C18 (Fig 9E and 9F). A possible explanation for this can be that *LsL* is mainly involved in the early vegetative growth and in the formation of axillary meristems [21,55], while our samples were taken later in vegetative growth with axillary meristems already established and developed to axillary buds. Similarly, *STM* also represents a gene that is involved in axillary meristem formation [23]. This could account for the lack of differential expression that was observed with *CmSTM* as there was a general increased expression in both C17 and C18.

## Conclusions

Our results showed that at floral transition in chrysanthemum genotype C17 the release from apical dominance with subsequent outgrowth of axillary buds coincided with an increased CK/IAA ratio. Conversely, in genotype C18, continuation of vegetative growth was accompanied by a decreased CK/IAA ratio. The expression of several genes, most notably *CmBRC1*, *CmDRM1* and *CmMAX1*, showed a differential expression pattern that coincided with the changes in CK/IAA ratio, with decreased expression at floral transition in C17 and increased expression with continued vegetative growth in C18. Furthermore, expression of auxin genes *CmTIR3*, *CmAXR2* and *CmIAA16* corresponded with a higher auxin status in C18 during vegetative growth, compared to C17. The expression of *CmBRC1*, *CmDRM1* and also *CmMAX1*, which was previously not yet reported in chrysanthemum, could be used as early indicators of bud outgrowth activity.

## Supporting Information

S1 Fig Virtual gel view and electropherograms of the Experion^™^ automated electrophoresis (Bio-Rad) for analysis of RNA quality in a subset of samples. Numbers 1 to 6 represent samples from C17. Numbers 7 to 12 represent samples from C18. Apex, bud and stem samples are included for both timepoints.
(PDF)

S2 Fig Functional domains on the isolated sequences in Chrysanthemum. Based on NCBI conserved domain search http://www.ncbi.nlm.nih.gov/Structure/cdd/wrpsb.cgi. The protein sequences for Chrysanthemum and Arabidopsis are presented together. For *CmARR1* and *CmTIR3*, the Chrysanthemum query sequence was too short to contain a functional domain. For these sequences an alignment to the Arabidopsis protein sequence (Clustal Omega http://www.ebi.ac.uk/Tools/msa/clustalo/) is provided below the conserved domains.
(PDF)

S3 Fig Hierarchical clustering. A dendrogram was constructed for both genotypes C17 and C18.
(TIF)

S4 Fig Canonical discriminant function analysis. Discriminant analysis was performed on the combined gene expression data of all genes that were common to all tissues. The analysis was done for both genotypes C17 and C18 at both time points together (A and D) and at the different time points: for C17: V1 (B) and T2 (C), for C18: V1 € and V2 (F). For all plots the percentage of variance explained by Function 1 and 2 are indicated as FCN1 and FCN2.
(TIF)

S1 Table MAX1 amino acid sequences that were used to make MAX1 degenerate primers. (PDF)

S2 Table Primers used for isolating reference and target genes from chrysanthemum cDNA. The accession number corresponds to the gene that was used for the BLAST search.
(PDF)

S3 Table RNA quality control. RNA concentration (ng/μl) and A260/280, A260/230 values of RNA isolated from the repeated V1 and V1/T2 shoot apex, stem and axillary bud samples of the chrysanthemum genotypes C17 and C18.
(PDF)

S4 Table Primers of reference and target genes used for RT-qPCR analysis. (PDF)

S5 Table Mean gene-specific PCR efficiencies, determined using LinRegPCR [61, 62], of reference and target genes used for RT-qPCR analysis. In batch 1 the expression of *CmBRC1*, *CmIPT*, *CmLsL*, *CmMAX1* and *CmMAX2* was analysed. Batch 2 was used for expression analysis of *CmRR1*, *CmAXR1*, *CmAXR2*, *CmAXR6*, *CmHK3a*, *CmHK3b*, *CmDRM1*, *CmIAA12*, *CmIAA16*, *CmMAX3*, *CmPIN1*, *CmSTM*, *CmTIR1* and *CmTIR3*.
(PDF)

S6 Table Reference target stabilities. M and CV values for the 3 reference genes that were used for normalisation in the RT-qPCR analysis of branching genes in apex and axillary bud tissue and in stem samples.
(PDF)

S7 Table Values of CK, IAA measurements from the repeated V1 and V1/T2 shoot apex, stem and axillary bud samples of the chrysanthemum genotypes C17 and C18. The fist tab shows IAA measurement and the total CK level, which is the sum of individual isoprenoid and aromatic cytokinins presented in the second tab.
(XLSX)

S8 Table Comparison of mean IAA and CK content between different zones of axillary buds and stem for C17 at V1 and T2 and for C18 at V1 and V2. Data are fold changes of hormone content (A-B = Zone-B/Zone-A) between mean (n = 3) IAA or CK contents and the significant difference between means by Kruskal-Wallis test is indicated by * (p-value<0.05).
(PDF)

S9 Table Comparison of mean IAA and CK content between different zones of axillary buds and stem for C17 between V1 and T2 and for C18 between V1 and V2. Data are fold changes (A-B = Zone-B/Zone-A) between mean (n = 3) IAA or CK contents and the significant difference between means by Kruskal-Wallis test is indicated by * (p-value<0.05).
(PDF)

S10 Table CNRQ gene expression data from RT-qPCR on the repeated V1 and V1/T2 shoot apex, stem and axillary bud samples of the chrysanthemum genotypes C17 and C18. (XLSX)

S11 Table Fold changes of gene expression levels in C17 between the different apex and axillary bud samples in V1 and T2. Data are fold changes (A-B = Zone-B/Zone-A) between mean CNRQ values (n = 3). The significant difference between means by Kruskal-Wallis test is indicated by * (p-value<0.05).
(PDF)

S12 Table Fold changes of gene expression in C17 between the different stem samples in V1 and T2. Data are fold changes (A-B = Zone-B/Zone-A) between mean CNRQ values (n = 3). The significant difference between means by Kruskal-Wallis test is indicated by * (p-value<0.05).
(PDF)

S13 Table Fold changes of gene expression between V1 and T2 for different samples of axillary bud and stem of C17. Data are fold changes (A-B = Zone-B/Zone-A) between mean CNRQ values (n = 3). The significant difference between means by Kruskal-Wallis test is indicated by * (p-value<0.05).
(PDF)

S14 Table Fold changes in gene expression in C18 between different apex and axillary bud samples for V1 and V2. Data are fold changes (A-B = Zone-B/Zone-A) between mean CNRQ values (n = 3). The significant difference between means by Kruskal-Wallis test is indicated by * (p-value<0.05).
(PDF)

S15 Table Fold changes in gene expression between stem samples in C18 V1 and V2. Data are fold changes (A-B = Zone-B/Zone-A) between mean CNRQ values (n = 3). The significant difference between means by Kruskal-Wallis test is indicated by * (p-value<0.05).
(PDF)

S16 Table Fold changes in gene expression between V1 and V2 of axillary bud and stem samples of C18. Data are fold changes (A-B = Zone-B/Zone-A) between mean CNRQ values (n = 3). The significant difference between means by Kruskal-Wallis test is indicated by * (p-value<0.05).
(PDF)
