# Supplementary figures and images for: Change in Auxin and Cytokinin Levels Coincides with Altered Expression of Branching Genes during Axillary Bud Outgrowth in Chrysanthemum
Source: PLoS One. 2016 Aug 24;11(8):e0161732. doi: 10.1371/journal.pone.0161732 (PMC4996534; doi:10.1371/journal.pone.0161732)

## Virtual Gel view

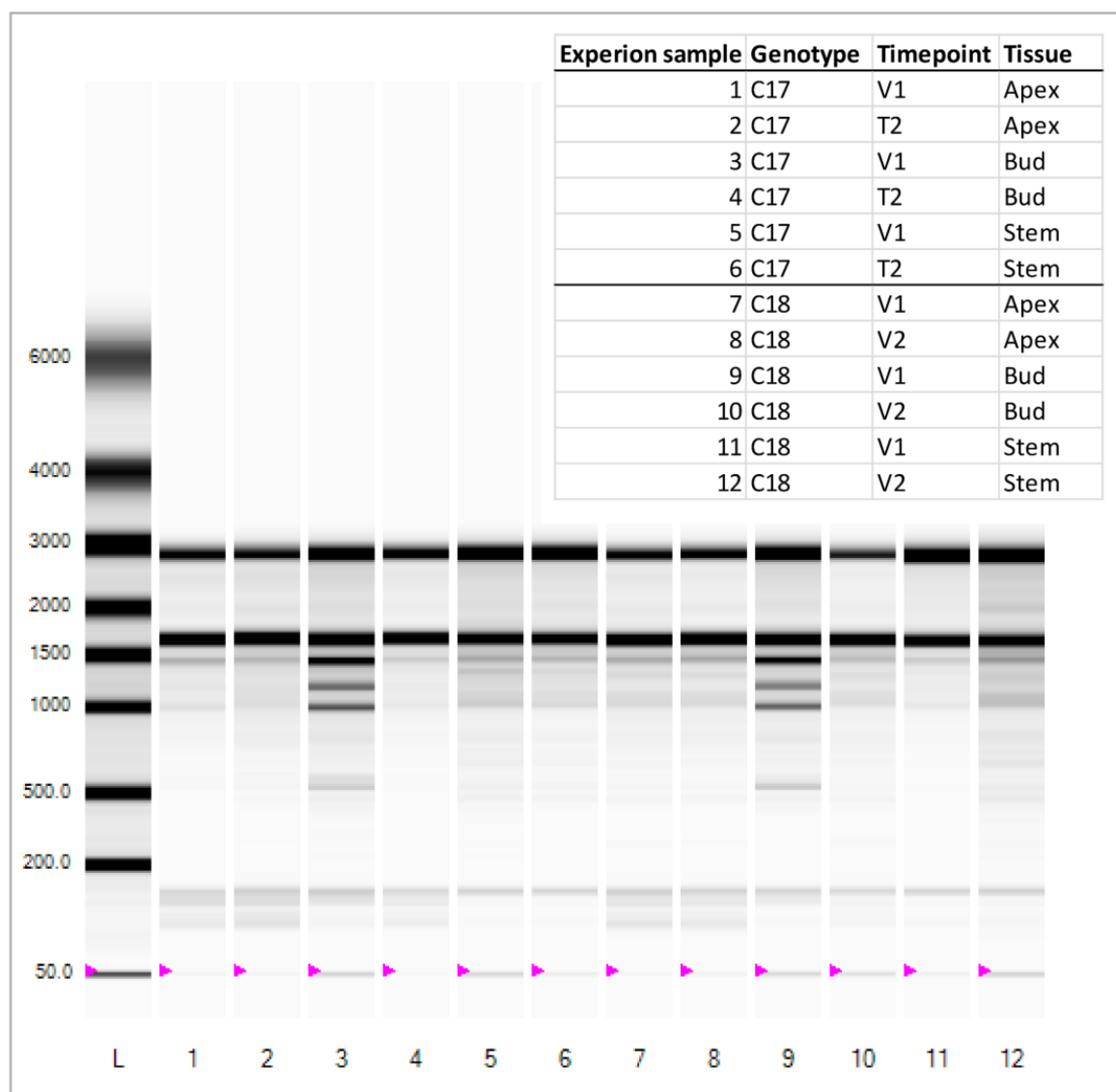

## Electropherogram

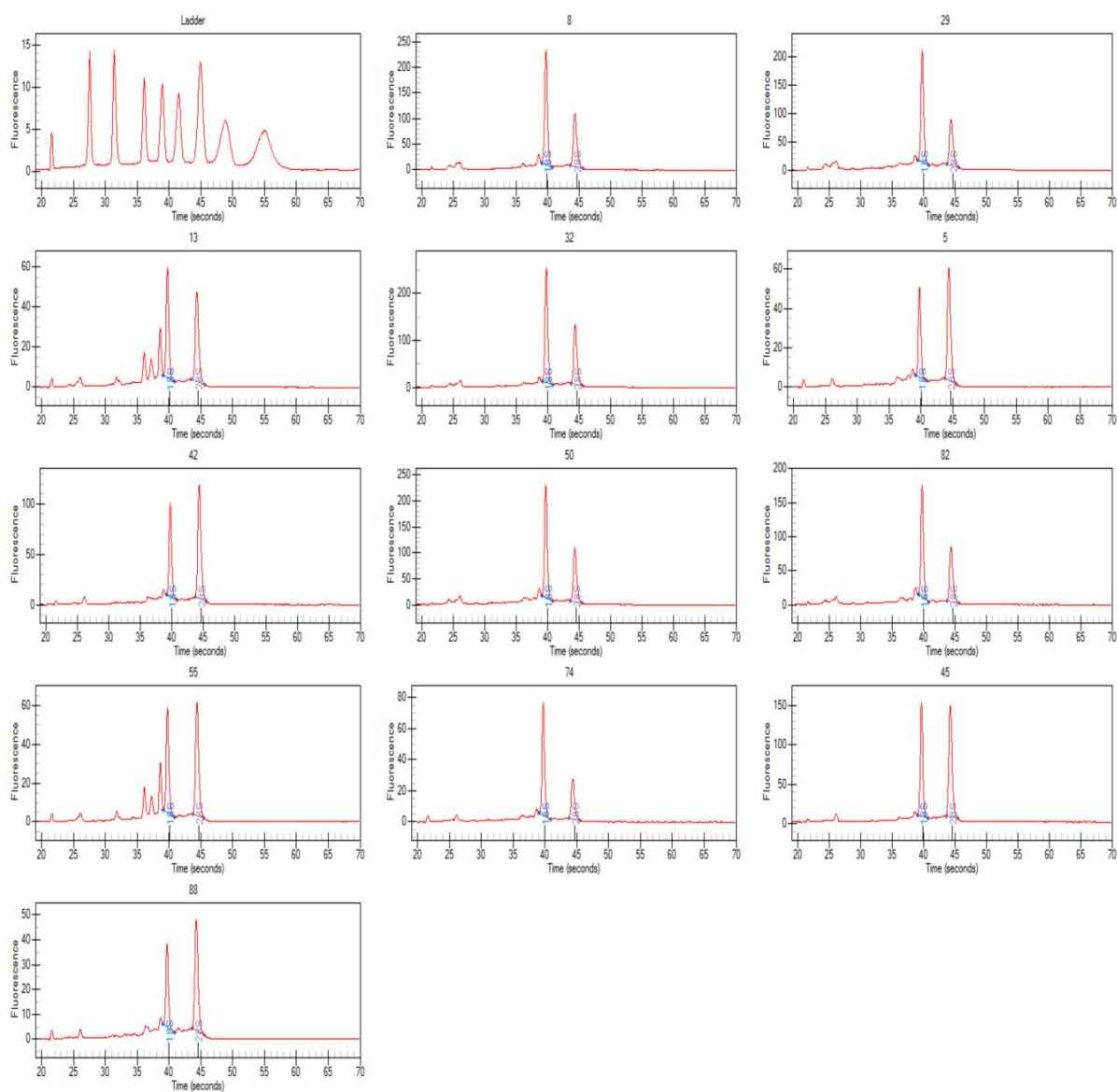

Supplement: S1 Fig — Numbers 1 to 6 represent samples from C17. Numbers 7 to 12 represent samples from C18. Apex, bud and stem samples are included for both timepoints. (PDF) [file pone.0161732.s001.pdf]

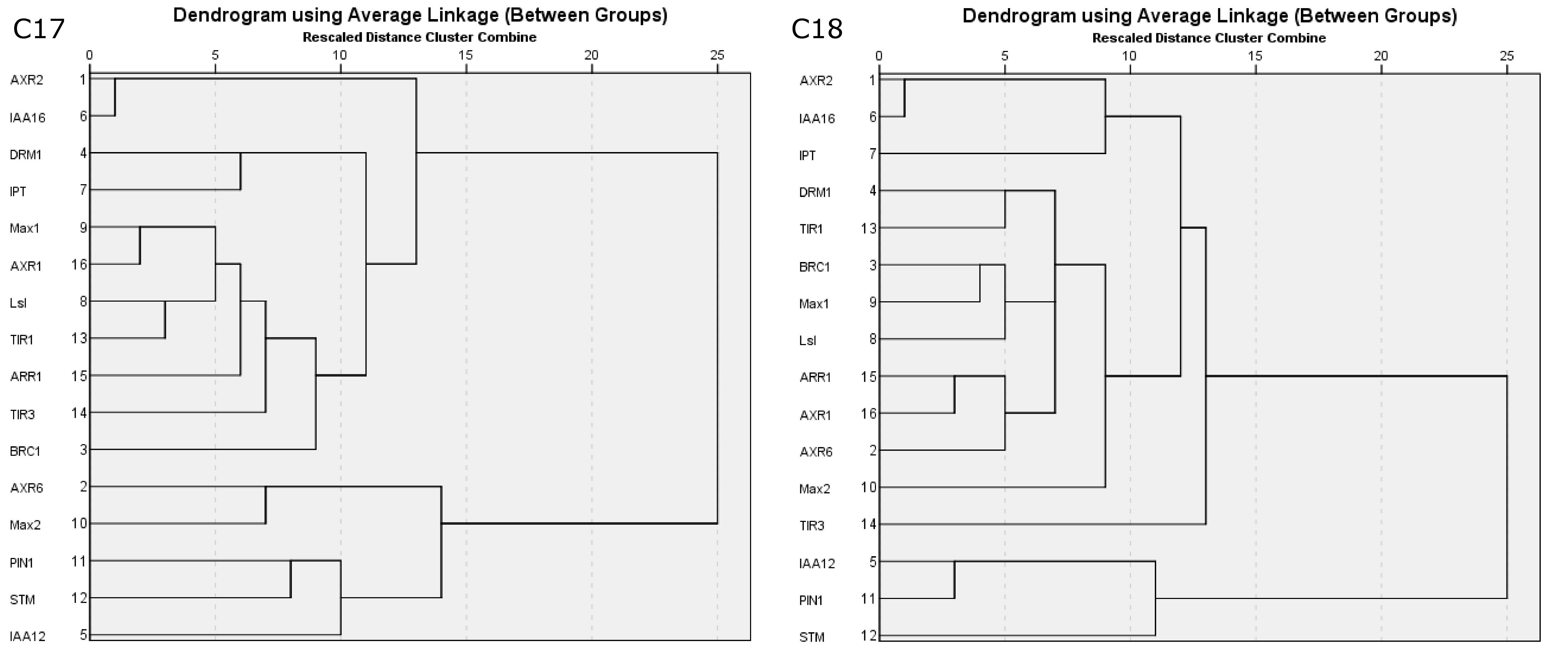

Supplement: S3 Fig — A dendrogram was constructed for both genotypes C17 and C18. (TIF) [file pone.0161732.s003.tif]

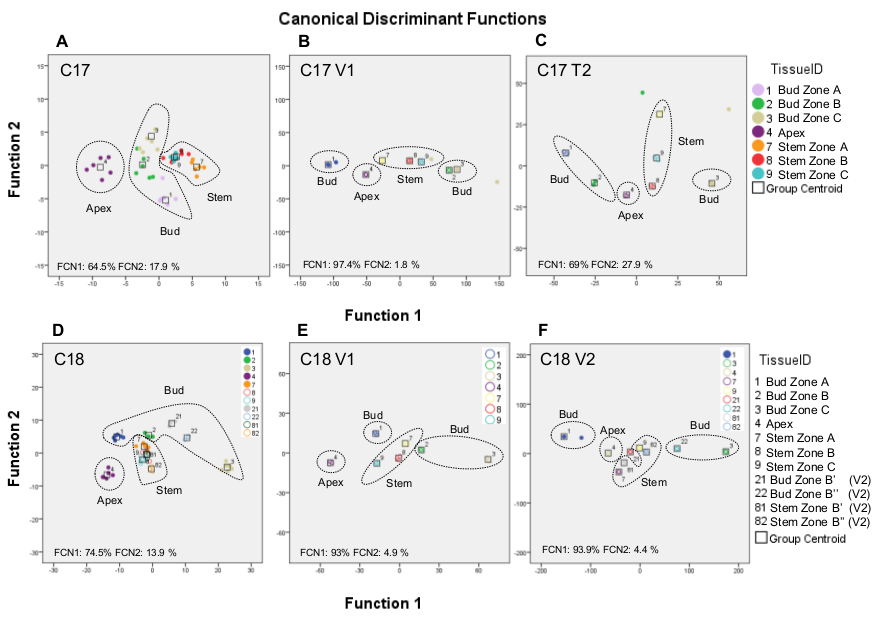

Supplement: S4 Fig — Discriminant analysis was performed on the combined gene expression data of all genes that were common to all tissues. The analysis was done for both genotypes C17 and C18 at both time points together (A and D) and at the different time points: for C17: V1 (B) and T2 (C), for C18: V1 € and V2 (F). For all plots the percentage of variance explained by Function 1 and 2 are indicated as FCN1 and FCN2. (TIF) [file pone.0161732.s004.tif]
