# Supplementary material for: Change in Auxin and Cytokinin Levels Coincides with Altered Expression of Branching Genes during Axillary Bud Outgrowth in Chrysanthemum
Source: PLoS One. 2016 Aug 24;11(8):e0161732. doi: 10.1371/journal.pone.0161732 (PMC4996534; doi:10.1371/journal.pone.0161732)
Supplement: S2 Fig — Based on NCBI conserved domain search http://www.ncbi.nlm.nih.gov/Structure/cdd/wrpsb.cgi. The protein sequences for Chrysanthemum and Arabidopsis are presented together. For CmARR1 and CmTIR3, the Chrysanthemum query sequence was too short to contain a functional domain. For these sequences an alignment to the Arabidopsis protein sequence (Clustal Omega http://www.ebi.ac.uk/Tools/msa/clustalo/) is provided below the conserved domains. (PDF) [file pone.0161732.s002.pdf]

Domain families on selected sequences

Q#1 - >CmARR1 ((Local ID))

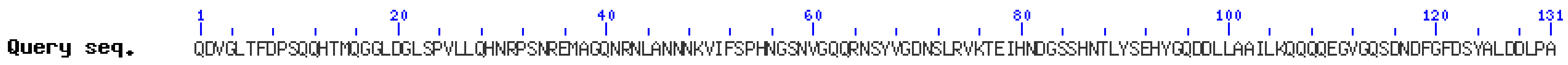

Q#1 - >ARR1_Ath gi|42 (ARR1_Ath gi|42564262|ref|NP_566561.2(Local ID))

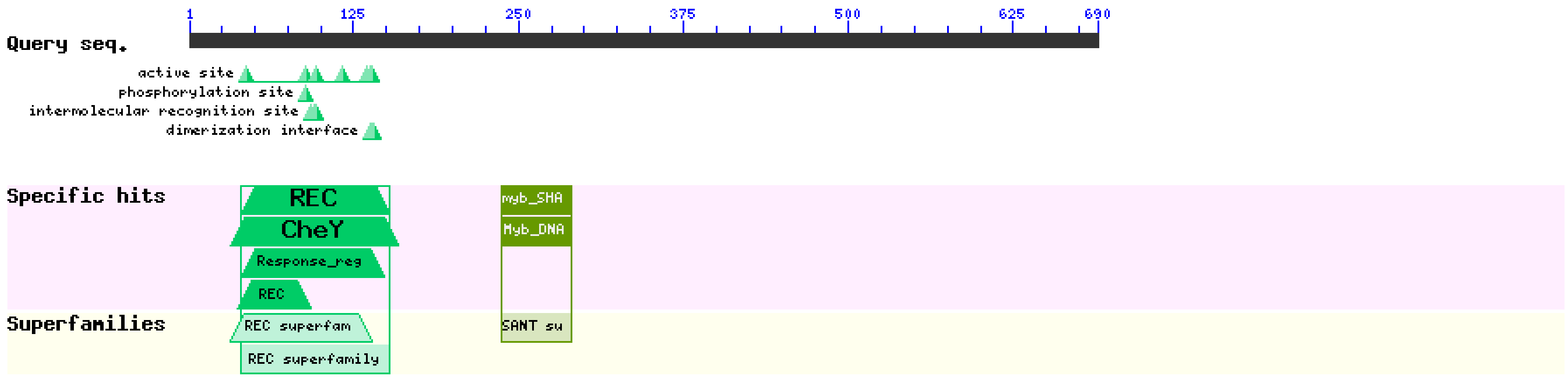

Q#2 - >CmAXR1 ((Local ID))

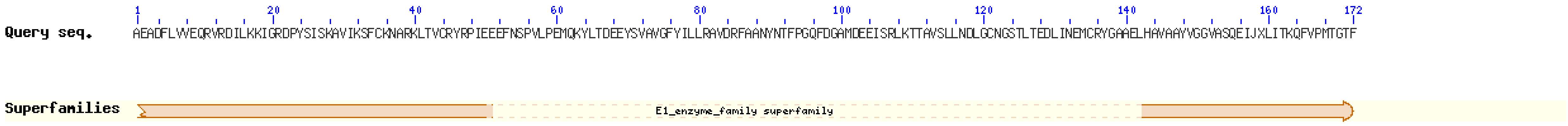

Q#2 - >AXR1_Ath gi|42 (AXR1_Ath gi|42571341|ref|NP_973761.1(Local ID))

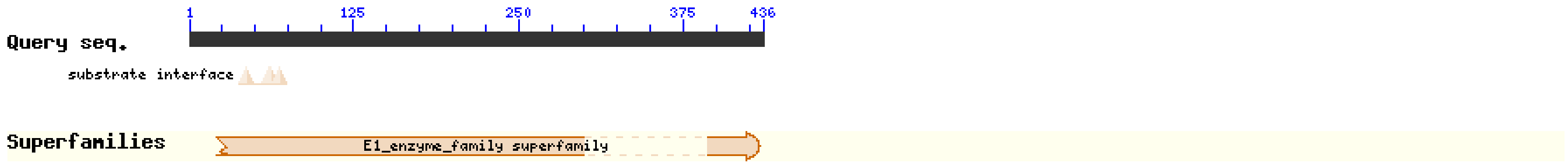

Q#3 - >CmAXR2 ((Local ID))

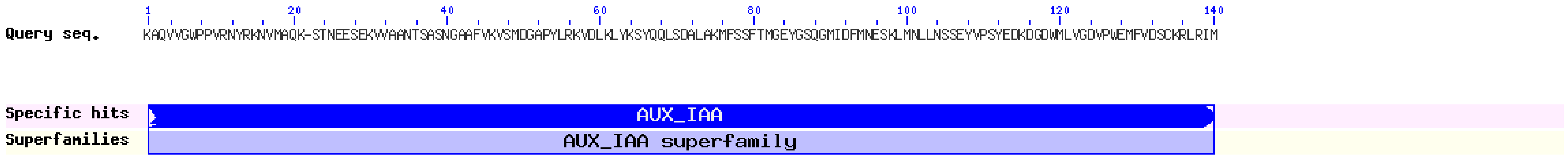

Q#3 - >AXR2_Ath gi|15 (AXR2_Ath gi|15228943|ref|NP_188945.1(Local ID))

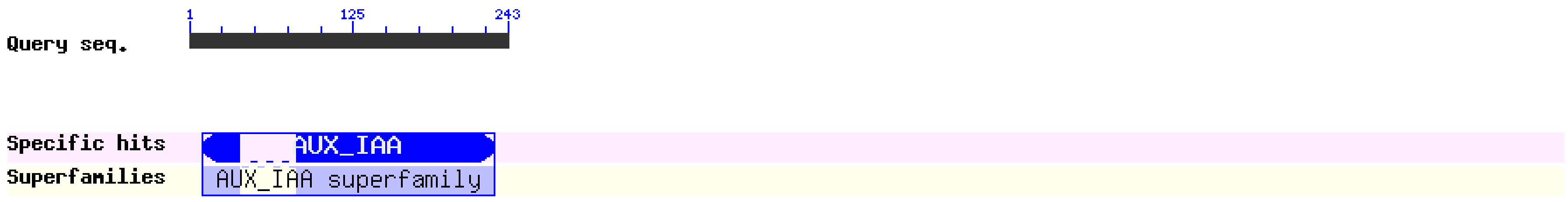

Q#4 - >CmAXR6 ((Local ID))

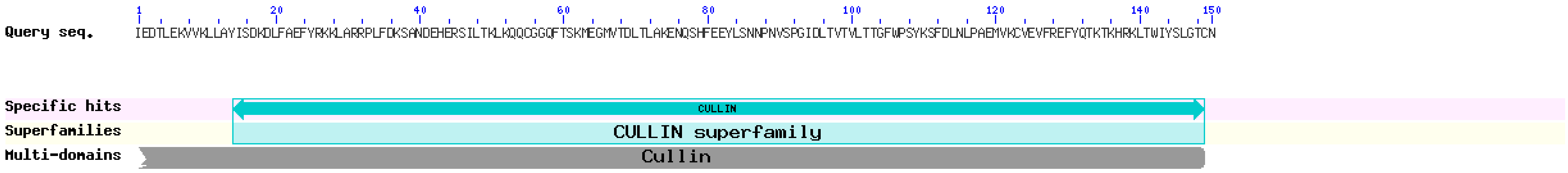

Q#4 - >AXR6_Ath gi|18 (AXR6_Ath gi|18411983|ref|NP_567243.1(Local ID))

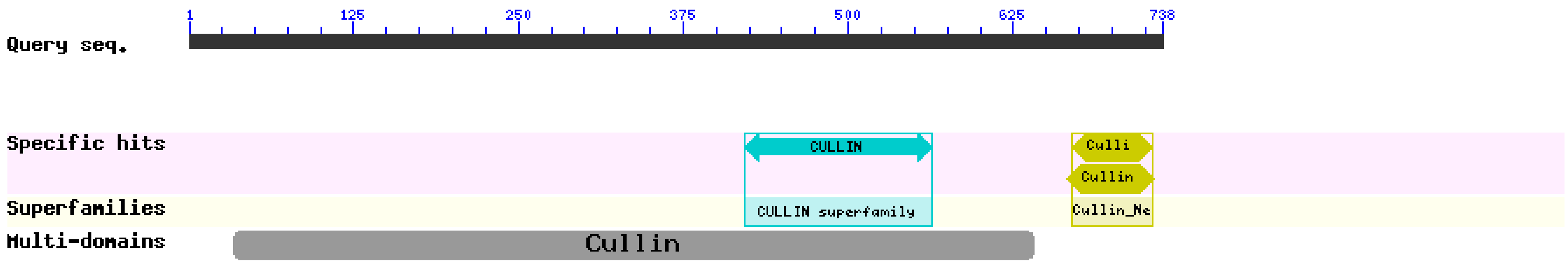

Q#5 - >CmIAA16 ((Local ID))

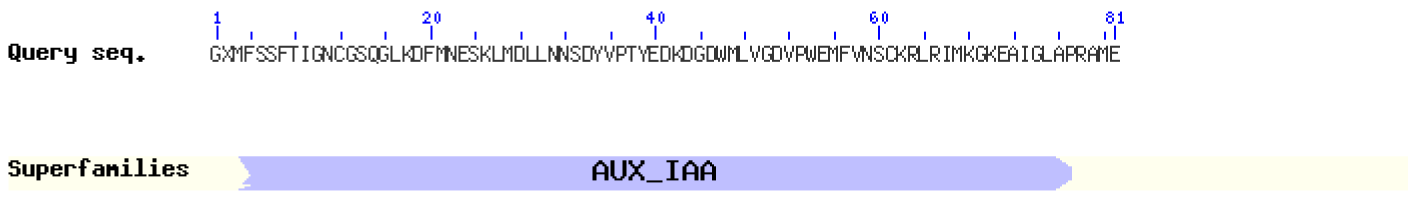

Q#5 - >IAA16_Ath gi|3 (IAA16_Ath gi|304322366|gb|ADL70670.1(Local ID))

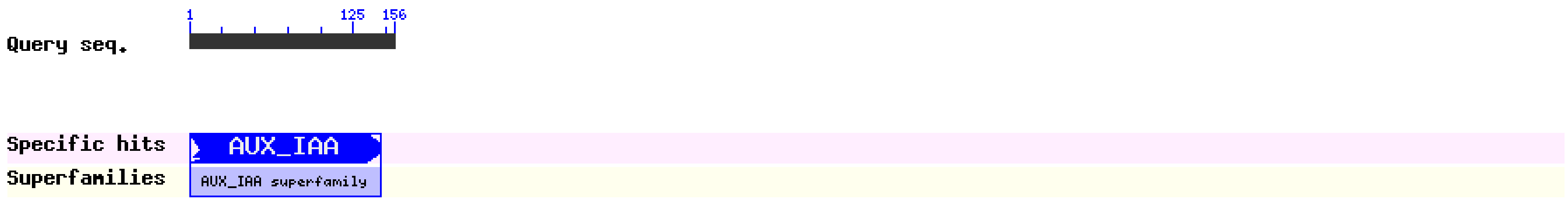

Q#6 - >CmHK3a ((Local ID))
Redundancy:

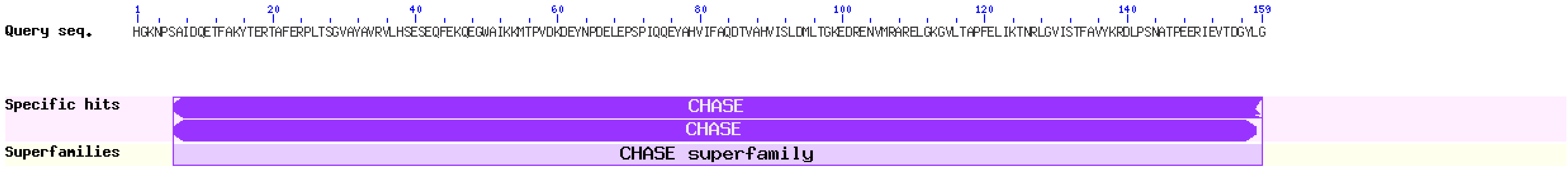

Q#6 - >CmHK3b ((Local ID))

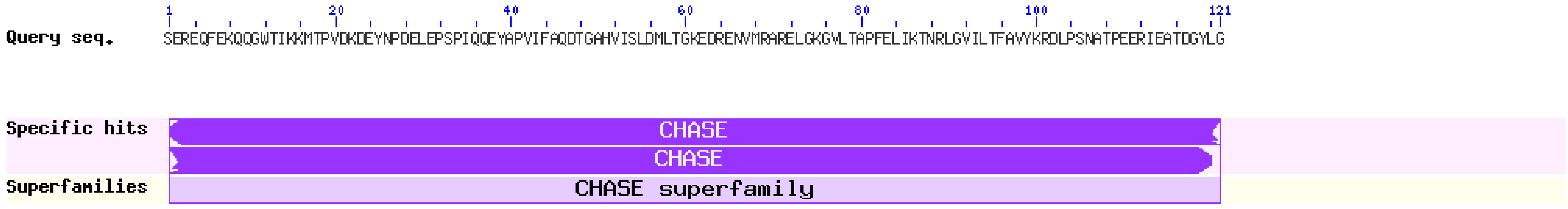

Q#6 - >AHK3_Ath gi|18 (AHK3_Ath gi|18396292|ref|NP_564276.1(Local ID))

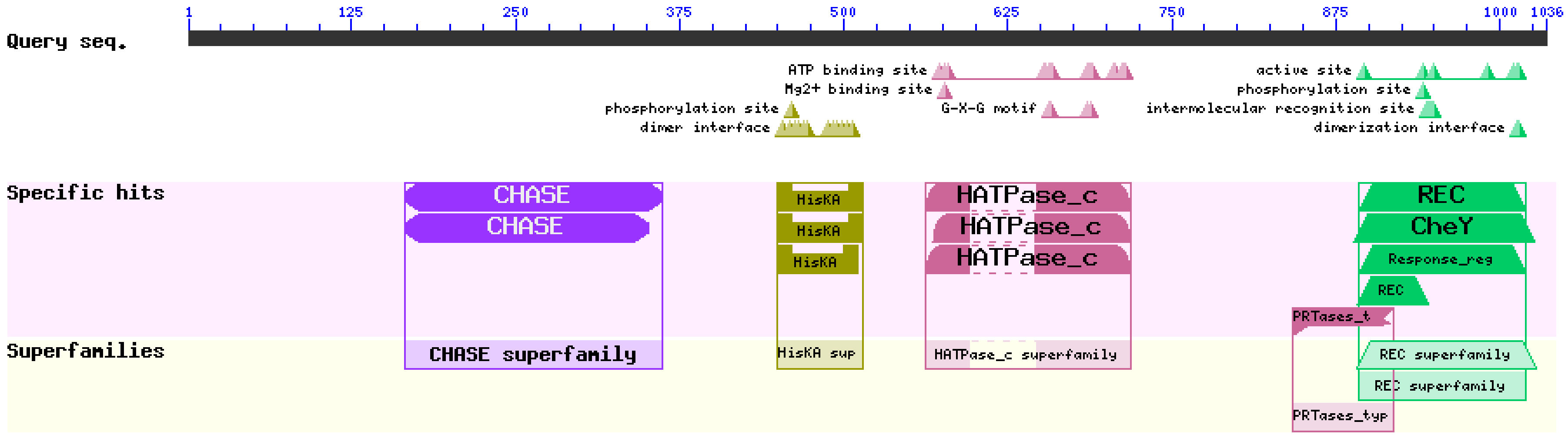

Q#7 - >DRM1 ((Local ID))

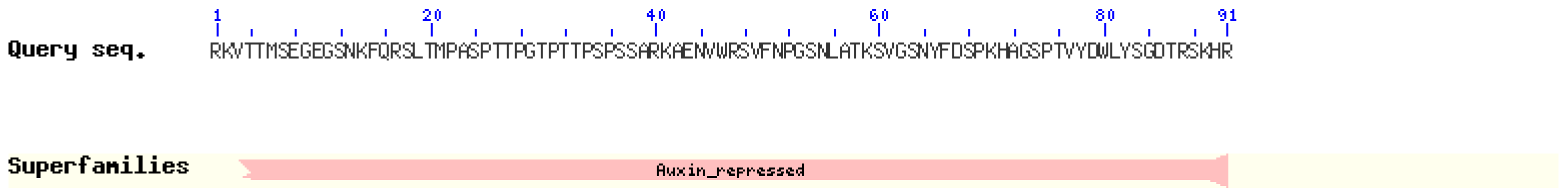

Q#8 - >DRM1_Ath gi|18 (DRM1_Ath gi|18396748|ref|NP_564305.1(Local ID))

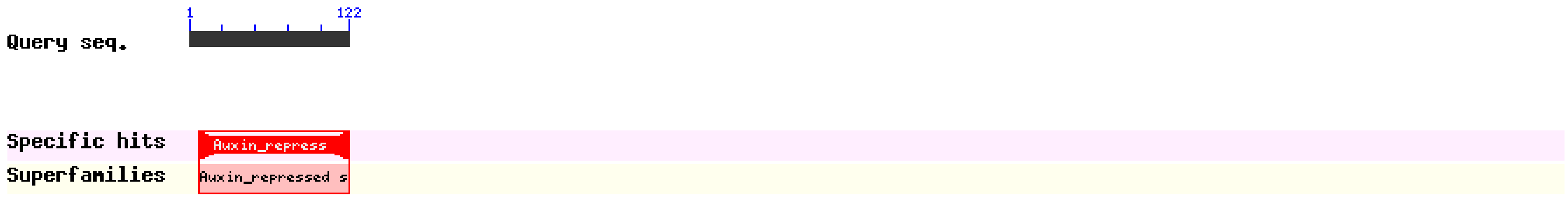

Q#9 - >CmIAA12 ((Local ID))

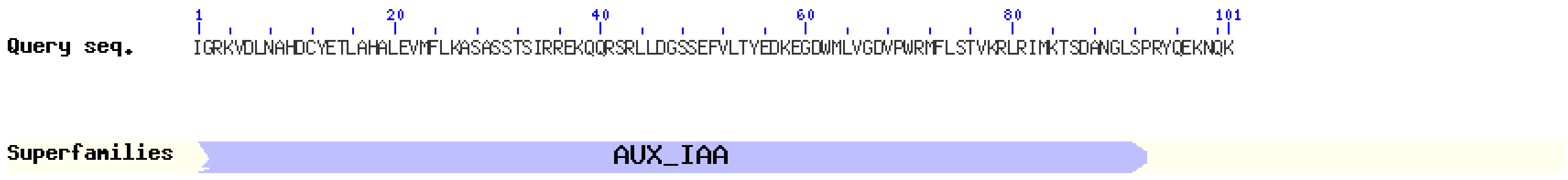

Q#9 - >IAA12_Ath gi|1 (IAA12_Ath gi|15219761|ref|NP_171949.1(Local ID))

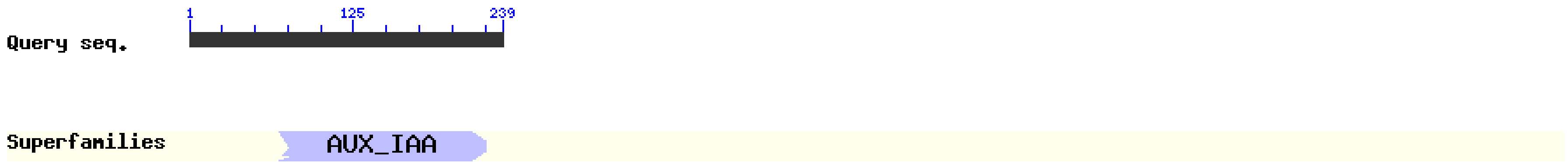

Q#10 - >CmMAX3 ((Local ID))

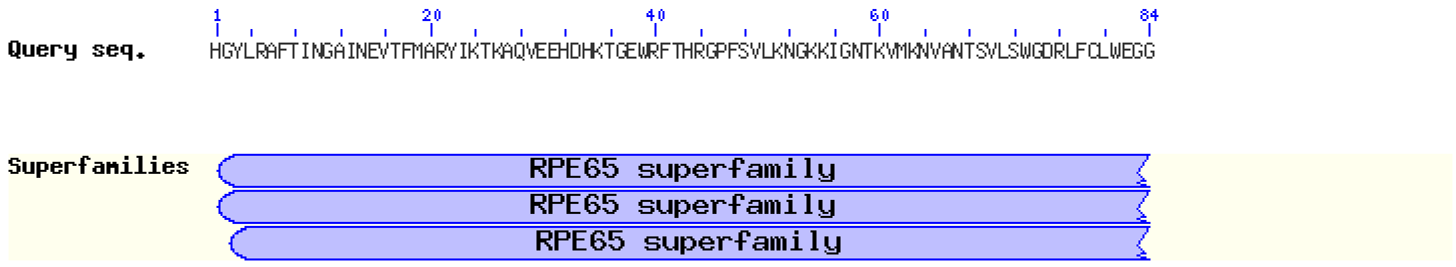

Q#10 - >MAX3_gi|148534 (MAX3_gi|148534385|gb|ABQ85317.1(Local ID))

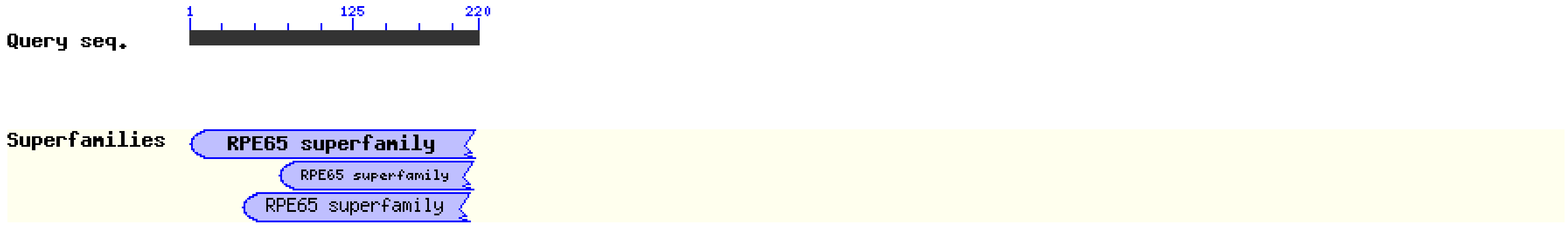

Q#11 - >CmPIN1 ((Local ID))

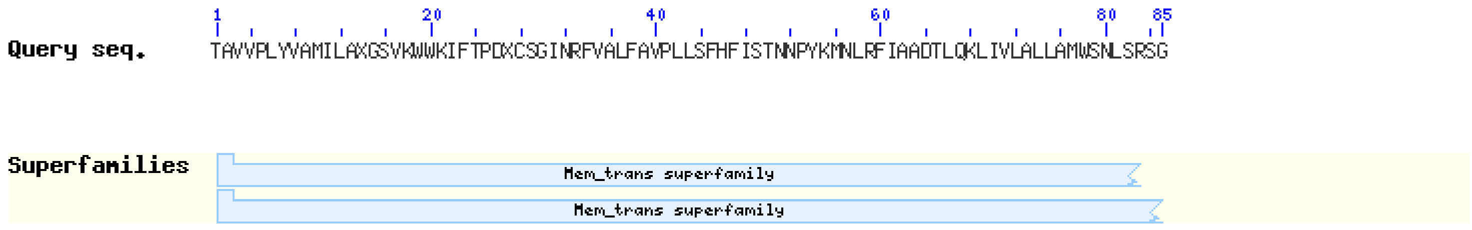

Q#11 - >PIN1_Athh gi|1 (PIN1_Athh gi|15219501|ref|NP_177500.1(Local ID))

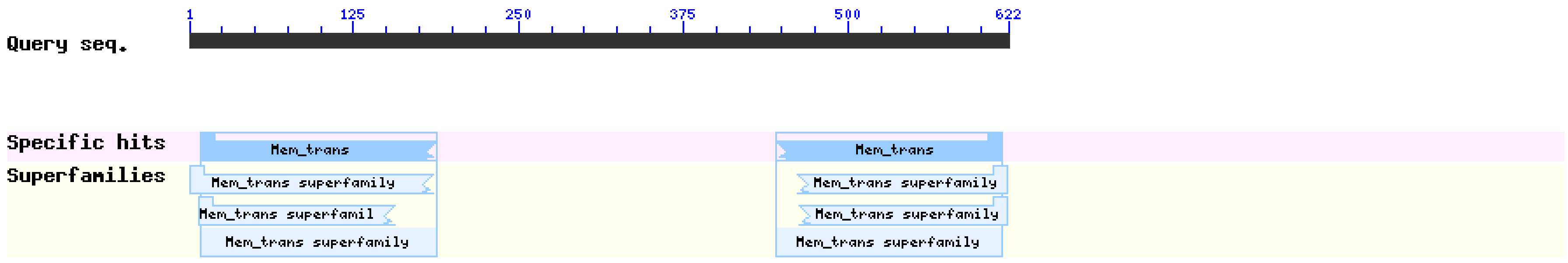

Q#12 - >CmSTM ((Local ID))

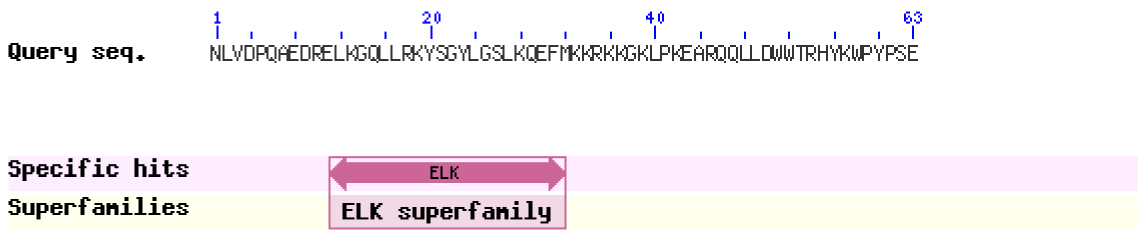

Q#12 - >STM_Ath gi|148 (STM_Ath gi|148729575|gb|ABR09190.1(Local ID))

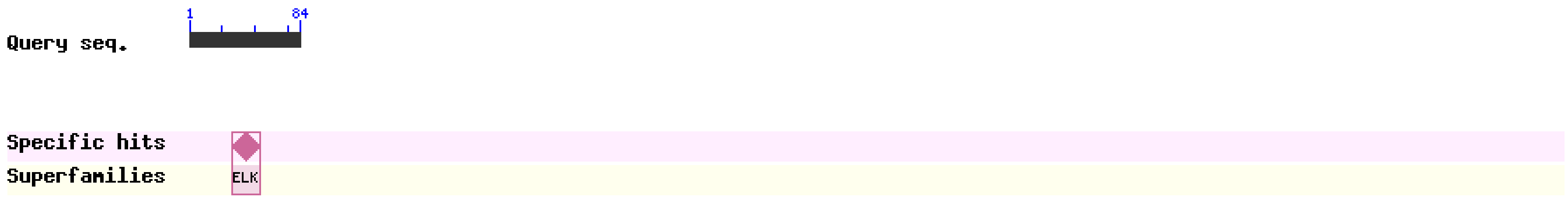

[Q#13 - >CmTIR1 ((Local ID))]

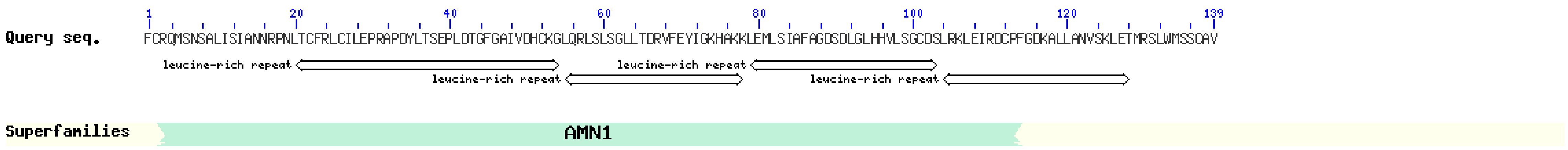

[Q#13 - >TIR1_Ath gi|28 (TIR1_Ath gi|284517076|gb|ADB92042.1(Local ID))]

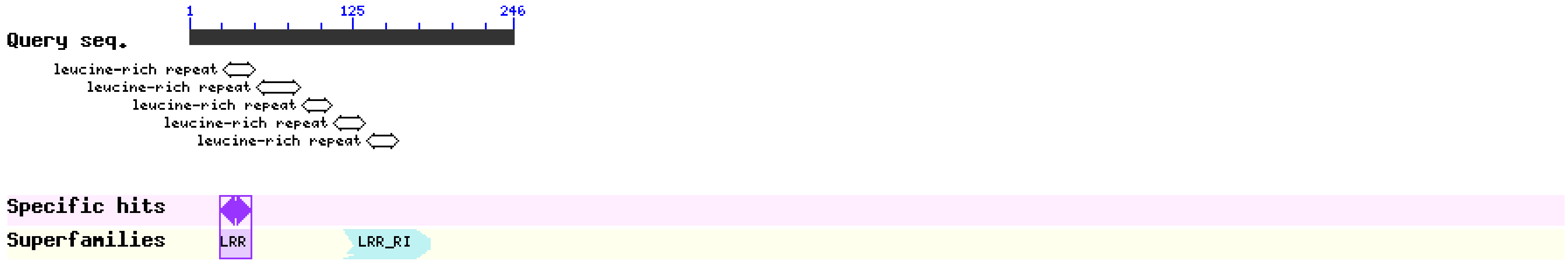

[Q#14 - >CmTIR3 ((Local ID))]

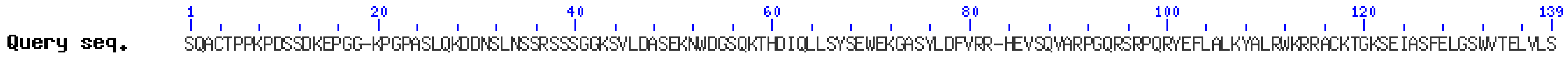

[Q#14 - >TIR3_Ath gi|10 (TIR3_Ath gi|1039016063|gb|ANM65563.1(Local ID))]

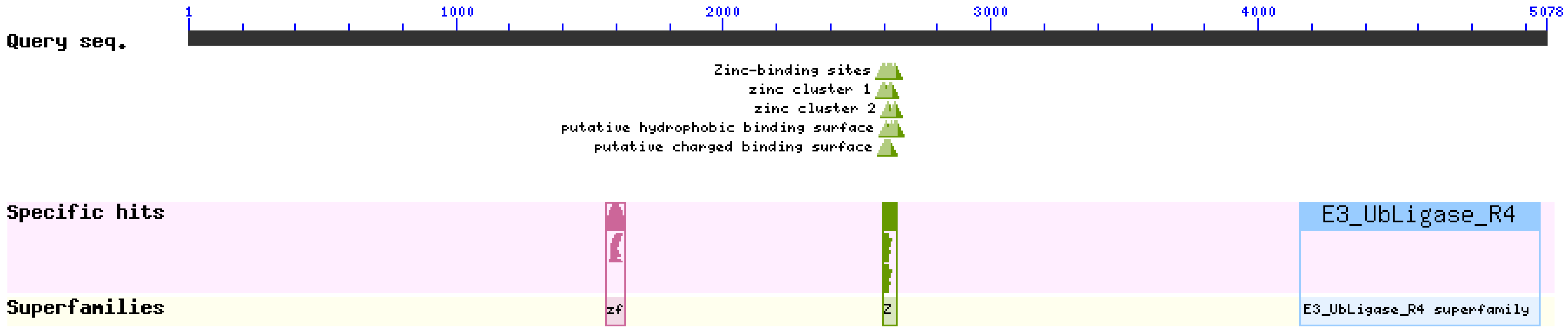

Clustal Omega protein sequence alignments of the *Arabidopsis thaliana ARR1* and *TIR3* with the query sequences from Chrysanthemum.

```
CmARR1       1 -----------------------------------------------------------------------------------------------
ARR1_Ath     1 MMNPSHGRGLGSAGGSSSGRNQGGGGETVVEMFPSGLRVLVVDDDPTCLMILERMLRTCLYEVTKCNRAEMALSLLRKNKHGFDIVISDVHMPDM

CmARR1       1 -----------------------------------------------------------------------------------------------
ARR1_Ath    96 DGFKLLEHVGLEMDLPVIMMSADDSKSVVLKGVTHGAVDYLIKPVRMEALKNIWQHVVRKRRSEWSVPEHSGSIEETGERQQQQHRGGGGGAAVS

CmARR1       1 -----------------------------------------------------------------------------------------------
ARR1_Ath   191 GGEDAVDDNSSSVNEGNNWRSSSRKRKDEEGEEQGDDKDEDASNLKKPRVVWSVELHQQFVAAVNQLGVEKAVPKKILELMNVPGLTRENVASHL

CmARR1       1 -----------------------------------------------------------------------------------------------
ARR1_Ath   286 QKYRIYLRRLGGVSQHQGNLNNSFMTGQDASFGPLSTLNGFDLQALAVTGQLPAQSLAQLQAAGLGRPAMVSKSGLPVSSIVDERSIFSFDNTKT

CmARR1       1 -----------------------------------------------------------------------------------------------
ARR1_Ath   381 RFGEGLGHHGQQPQQQPQMNLLHGVPTGLQQQLPMGNRMSIQQQIAAVRAGNSVQNNGMLMPLAGQQSLPRGPPPMLTSSQSSIRQPMLSNRISE

CmARR1       1 ---------------------------------------------------------------------------------------------QD
ARR1_Ath   476 RSGFSGRNNIPESSRVLPTSYTNLTTQHSSSSMPYNNFQPELPVNSFPLASAPGISVPVRKATSYQEEVNSSEAGFTTPSYDMFTTRQNDWDLRN

CmARR1       3 VGLTFDPSQQHTMQGG--LDGLS-PVLLQHNRPSNREMAGQNRNLANNNKVIFSPHNGSNVGQQRNSYVG--DNSLRVKTEIHNDGSSHNTLYSE
ARR1_Ath   571 IGIAFDSHQDSESAAFSASEAYSSSSMSRHNTTVAATEHGRNHQ---------QPPSG--MVQHHQVYADGNGGSVRVKSERVATDTA-TMAFHE

CmARR1      93 HY-GQDDLLAAILKQQQQEGVGQSDNDFGFDSYALDDLPA
ARR1_Ath   654 QYSNQEDLMSALLK---QEGIAPVDGEFDFDAYSIDNIPV
```

```
CmTIR3      1 ------------------------------------------------------------------------------------------------------------------------
TIR3_Ath 1921 QYFTLPNDMIVDATLFVASRGRVFLLVLSEQGNLYRFELSWGGNAGATPLKEIVQIMGKDVTGKGSSVYFSPTYRLLFISYHDGSSFMGRLSSDATSLTDTSGMFEEESDCKQRVAGLHR

CmTIR3      1 ------------------------------------------------------------------------------------------------------------------------
TIR3_Ath 2041 WKELLAGSGLFICFSSVKSNAVLAVSLRGDGVCAQNLRHPTGSSSPMVGITAYKPLSKDNVHCLVLHDDGSLQIYSHVRSGVDTDSNFTAEKVKKLGSKILNNKTYAGAKPEFPLDFFER

CmTIR3      1 ------------------------------------------------------------------------------------------------------------------------
TIR3_Ath 2161 AFCITADVRLGSDAIRNGDSEGAKQSLASEDGFIESPSPVGFKISVSNPNPDIVMVGIRMHVGTTSASSIPSEVTIFQRSIKMDEGMRCWYDIPFTVAESLLADEDVVISVGPTTSGTAL

CmTIR3      1 ------------------------------------------------------------------------------------------------------------------------
TIR3_Ath 2281 PRIDSLEVYGRAKDEFGWKEKMDAVLDMEARVLGHGLLLPGSSKKRALAQSASMEEQVIADGLKLLSIYYSVCRPRQEVVLSELKCKQLLETIFESDRETLLQTTACRVLQSVFPRKEIY

CmTIR3      1 ------------------------------------------------------------------------------------------------------------------------
TIR3_Ath 2401 YQVKDTMRLLGVVKVTSILSSRLGILGTGGSIVEEFNAQMRAVSKVALTRKSNFSVFLEMNGSEVVDNLMQVLWGILESEPLDTPTMNNVVMSSVELIYSYAECLASQGKDTGVHSVAPA

CmTIR3      1 ------------------------------------------------------------------------------------------------------------------------
TIR3_Ath 2521 VQLLKALMLFPNESVQTSSSLAISSRLLQVPFPKQTMLTTDDLVDNVTTPSVPIRTAGGNTHVMIEEDSITSSVQYCCDGCSTVPILRRRWHCTVCPDFDLCEACYEVLDADRLPPPHTR

CmTIR3      1 ------------------------------------------------------------------------------------------------------------------------
TIR3_Ath 2641 DHPMTAIPIEVESLGADTNEIQFSADEVGISNMLPVVTSSIPQASTPSIHVLEPGESAEFSASLTDPISISASKRAVNSLILSEFLQELSGWMETVSGVQAIPVMQLFYRLSSAIGGAFM

CmTIR3      1 ------------------------------------------------------------------------------------------------------------------------
TIR3_Ath 2761 DSSKPEEISLDKLIKWLLGEINLSKPFAASTRSSLGEIVILVFMFFTLMLRSWHQPGSDGSSSKLGGSTDVHDRRIVQSSTVVATQSSLHVQERDDFASQLVRACSCLRNQEFVNYLMNI

CmTIR3      1 ------------------------------------------------------------------------------------------------------------------------
TIR3_Ath 2881 LQQLVHVFKSRAANVEARGSSSGSGCGAMLTVRRDLPAGNYSPFFSDSYAKAHRADIFVDYHRLLLENVFRLVYTLVRPEKQEKMGEKEKVYRNASSKDLKLDGFQDVLCSYINNPHTAF

CmTIR3      1 ------------------------------------------------------------------------------------------------------------------------
TIR3_Ath 3001 VRRYARRLFLHLCGSKTQYYSVRDSWQFSNEVKNLYKHVEKSGGFENNVSYERSVKIVKSLSTIAEVAVARPRNWQKYCLRHGDFLSFLLNGVFHFAEESVIQTLKLLNLAFYQGKDVSS

CmTIR3      1 ------------------------------------------------------------------------------------------------------------------------
TIR3_Ath 3121 SVQKAEATEVVTGSNRSGSQSVDSKKKKKGEDGHDSGLEKLYVDMEGVVDIFSANCGDLLRQFIDFFLLEWNSSSVRTEAKSVIYGLWHHGRHSFKESLLAALLQKVRYLPAYGQNIVEY

CmTIR3      1 ------------------------------------------------------------------------------------------------------------------------
TIR3_Ath 3241 TELVSLLLDKAPENNSKQAINELVDRCLNPDVIRCFFETLHSQNELIANHPNSRIYSTLGNLVEFDGYYLESEPCVACSSPDVPYSKMKLESLKSETKFTDNRIIVKCTGSYTIQSVTMN

CmTIR3      1 ------------------------------------------------------------------------------------------------------------------------
TIR3_Ath 3361 VHDARKSKSVKVLNLYYNNRPVSDLSELKNNWSLWKRAKSCHLSFNQTELKVEFPIPITACNFMIELDSFYENLQALSLEPLQCPRCSRPVTDKHGICSNCHENAYQCRQCRNINYENLD

CmTIR3      1 ------------------------------------------------------------------------------------------------------------------------
TIR3_Ath 3481 SFLCNECGYSKYGRFEFNFMAKPSFIFDNMENDEDMKKGLAAIESESENAHKRYQQLLGFKKPLLKIVSSIGETEMDSQHKDTVQQMMASLPGPSCKINRKIALLGVLYGEKCKAAFDSV

CmTIR3      1 ------------------------------------------------------------------------------------------------------------------------
TIR3_Ath 3601 SKSVQTLQGLRRVLMSYLHQKNSNFSSGASRCVVSKTPNNCYGCATTFVTQCLEILQVLSKHPRSRKQLVAAGILSELFENNIHQGPKTARAQARAALSTFSEGDLSAVNELNNLVQKKI

CmTIR3      1 ----------------------------------------------------------------------SQACTPPKPDSSDKEPGG-KPGPASLQKDDNSLNSSRSSSGGKSVLDASE
TIR3_Ath 3721 MYCLEHHRSMDIALATREEMLLLSEVCSLTDEFWESRLRLVFQLLFSSIKLGAKHPAISEHIILPCLKIISVACTPPKPDTAEKEQTMGKSAPAVQEKDENAAGVIKYS------SESEE

CmTIR3     50 KNWDGSQKTHDIQLLSYSEWEKGASYLDFVRRHEVSQ--VARPGQRSRPQRYEFLALKYALRWKRRACKTGKSEIASFELGSWVTELVLS------------------------------
TIR3_Ath 3835 NNLNVSQKTRDIQLVSYLEWEKGASYLDFVRRQYKASQSIRGASQKSRTHRSDFLALKYTLRWKRRSSRTSKGGLQAFELGSWVTELILSACSQSIRSEMCTLISLLAAQSSPRRYRLIN

CmTIR3        ------------------------------------------------------------------------------------------------------------------------
TIR3_Ath 3955 LLIGLLPATLAAGESSAEYFELLFKMIETQDALLFLTVRGCLTTICKLISQEVGNIESLERSLQIDISQGFTLHKLLELLGKFLEVPNIRSRFMRDNLLSHVLEALIVIRGLIVQKTKLI

CmTIR3        ------------------------------------------------------------------------------------------------------------------------
TIR3_Ath 4075 NDCNRRLKDLLDGLLLESSENKRQFIRACVSGLQTHAEENKGRTCLFILEQLCNLICPSKPEAVYMLILNKSHTQEEFIRGSMTKNPYSSAEIGPLMRDVKNKICQQLDLLGLLEDDYGM

CmTIR3        ------------------------------------------------------------------------------------------------------------------------
TIR3_Ath 4195 ELLVAGNIISLDLSIAQVYELVWKKSNQSSTSLTNSALLASNAAPSRDCPPMTVTYRLQGLDGEATEPMIKELEEDREESQDPEIEFAIAGAVREYGGLEILLDMIKSLQDDFKSNQEEM

CmTIR3        ------------------------------------------------------------------------------------------------------------------------
TIR3_Ath 4315 VAVLDLLNHCCKIRENRRALLRLGALSLLLETARRAFSVDAMEPAEGILLIVESLTLEANESDSISAAQSALTVSNEETGTWEQAKKIVLMFLERLSHPSGLKKSNKQQRNTEMVARILP

CmTIR3        ------------------------------------------------------------------------------------------------------------------------
TIR3_Ath 4435 YLTYGEPAAMEALIEHFSPYLQNWSEFDQLQQRHEEDPKDDSIAQQAAKQRFTVENFVRVSESLKTSSCGERLKDIVLENGIIAVAVKHIKEIFAITGQTGFKSSKEWLLALKLPSVPLI

CmTIR3        ------------------------------------------------------------------------------------------------------------------------
TIR3_Ath 4555 LSMLRGLSMGHLPTQTCIDEGGILTLLHALEGVSGENDIGARAENLLDTLADKEGKGDGFLGEKVRALRDATKDEMRRRALRKREELLQGLGMRQELSSDGGERIVVSQPILEGFEDVEE

CmTIR3        ------------------------------------------------------------------------------------------------------------------------
TIR3_Ath 4675 EEDGLACMVCREGYKLRPSDLLGVYSYSKRVNLGVGNSGSARGECVYTTVSYFNIIHFQCHQEAKRADAALKNPKKEWEGAMLRNNESLCNSLFPVKGPSVPLAQYLRYVDQYWDNLNAL

CmTIR3        ------------------------------------------------------------------------------------------------------------------------
TIR3_Ath 4795 GRADGSRLRLLTYDIVLMLARFATGASFSADCRGGGRDSNSRFLPFMFQMARHLLDQGGPVQRTNMARSVSSYISSSSTSTATAPSSDSRPLTPGSQLSSTGTEETVQFMMVNSLLSESY

CmTIR3        ------------------------------------------------------------------------------------------------------------------------
TIR3_Ath 4915 ESWLQHRRVFLQRGIYHTFMQHAHGRVASRAAEPTSSGGKTQDAETLTGDELLSIVKPMLVYTGMIEQLQQLFKPKKPVHIEPIKKEGTSSGVELEPWEIVMKEKLLNVKEMIGFSKELI

CmTIR3        --------------------------------------------
TIR3_Ath 5035 SWLDEINSATDLQEAFDIVGVLADVLSEGVTQCDQFVRSAIDKD
```
