# Supplementary material for: Change in Auxin and Cytokinin Levels Coincides with Altered Expression of Branching Genes during Axillary Bud Outgrowth in Chrysanthemum
Source: PLoS One. 2016 Aug 24;11(8):e0161732. doi: 10.1371/journal.pone.0161732 (PMC4996534; doi:10.1371/journal.pone.0161732)
Supplement: S1 Table — (PDF) [file pone.0161732.s005.pdf]

**Gene**	**Organism**	**Accession number**
MAX1	Arabidopsis thaliana	AT2G26170.1
MAX1	Solanum lycopersicum	XP_004245085
MAX1	Populus trichocarpa	XP_006372016
MAX1	Glycine max	XP_003549345
MAX1	Medicago truncatula	XP_003603162
MAX1	Vitis vinifera	XP_002279086
MAX1	Ricinus communis	XP_002516084
