# Supplementary material for: Change in Auxin and Cytokinin Levels Coincides with Altered Expression of Branching Genes during Axillary Bud Outgrowth in Chrysanthemum
Source: PLoS One. 2016 Aug 24;11(8):e0161732. doi: 10.1371/journal.pone.0161732 (PMC4996534; doi:10.1371/journal.pone.0161732)
Supplement: S2 Table — The accession number corresponds to the gene that was used for the BLAST search. (PDF) [file pone.0161732.s006.pdf]

Type	Gene	Acc. Nr.	Forward primer	Reverse primer
Reference genes	***CmUBC***	DK938098	AAAATGCAGGCATCAAGAGC	ACCCGATCTGAGAAGATTGC
	CmUBQ10	DK943260	GGCTGCAGGAAAGACCATTA	GCACAAGATGGAGGGTTGAT
	CmEF1α	DK939647	CCGGCAAGTCTACAACCACT	ACACCAAGGGTGAAAGCAAG
	CmACT2	DK942730	AATGGTACCGGAATGGTGAA	AGACGAAGAAGGCATGAGGA
	CmATUB	DK939980	GCCGGTATTCAAGTCGGTAA	CGCTCCAACAGAAGAGAACC
	CmCACS	DK938195	CATGGAACAATATGGGCATT	TGAGTGCAGAAGTGGAGTTGA
	CmEXP5	DK940274	ACCACAACCACCACCACTCT	CATGCCAAGACGCTTACCTT
	CmEXP6	DK936679	ACATGCCAAAAGTCGAGGAC	CCCCTATGCACAAGGTGTCT
	CmPGK	DK940990	CCATCTGTTGCTGGTTTCCT	CACCCATCTGGGATAGCAGT
	CmPSAA	JG700150	GGCACAGTCCTCCCAAGTAA	CCAATAACCACGACCGCTAA
	CmBTUB	AB608732	GGTGCCGAGCTAGTTGACTC	GAGTTGAGTTGACCCGGAAA
	CmMTP	AB542716.1	CAACTCCCAAAACCCTCAAA	GAAGCTCTCCTGCCATCAAC
	CmHH3	DK937563	CACACCTTCACCGAAAATGA	GAAGCAACTGGCTACCAAGG
Target genes	***CmMAX1***	KT124645	GGICCIATHTTYMGITTYCARATG	ACICCRAAIGCIGCYTGICCTAC
	CmMAX3	UN31745	CCACCTTCCCACAAACAAAA	CATGGCTATCTTAGAGCGTTCA
	CmDRM1	UN71012	CGGCATACAACAACAACGAT	CAAGCTACGCAAAGTCACCA
	CmPIN1	UN40485	GCCACTCCTACTGAGGTTGC	ACAGCAGTGGTGCCATTGTA
	CmAXR1	UN93500	TGAAAGTTCCCGTCATAGGC	GGCTGAGGCTGATTTTCTTG
	CmTIR1	UN83015	CTGACTGCGCATGAAGACAT	CTTCTGCCGACAGATGTCAA
	CmIAA12	UN61458	CGAATTGAGGCAAAAGGGTA	TGACGATTGGGAGGAAAGTC
	CmRR1	UN66918	TTTGGCTATTCGGATGGAAG	CAGGATGTCGGTTTGACCTT
	CmHK3a	UN55584	CCACCAAGATACCCGTCAGT	TCATGGGAAGAACCCTTCTG
	CmSTM	UN85005	CCAGTTGTTTATCTGCTTCTGGT	CAATCTCGTAGATCCTCAAGCTG
	CmTIR3	UN96999	CAGCATTTTCACCAGCAGAA	TTCACAAGCATGCACTCCTC
	CmAXR2	UN43016	TCATGATTCGAAGTCGCTTG	CCAAGGCTCAAGTGGTAGGA
	CmIAA16	UN00818	TCCAAGTTCCAAATGCACAA	GGGCAACATGTTCAGTTCCT
	CmAXR6	UN90522	TGTTGCAGGTACCCAATGAA	CATTGAGGACACGCTTGAGA
