# Supplementary material for: Change in Auxin and Cytokinin Levels Coincides with Altered Expression of Branching Genes during Axillary Bud Outgrowth in Chrysanthemum
Source: PLoS One. 2016 Aug 24;11(8):e0161732. doi: 10.1371/journal.pone.0161732 (PMC4996534; doi:10.1371/journal.pone.0161732)
Supplement: S3 Table — RNA concentration (ng/μl) and A260/280, A260/230 values of RNA isolated from the repeated V1 and V1/T2 shoot apex, stem and axillary bud samples of the chrysanthemum genotypes C17 and C18. (PDF) [file pone.0161732.s007.pdf]

Sample nr.	Genotype	Timepoint	Tissue	Position	Repeat	ng/ul	260/280	260/230
1	C17	V1	Apex	Apex	I	345,52	1,99	2,12
8	C17	V1	Apex	Apex	II	404,06	2,02	2,16
15	C17	V1	Apex	Apex	III	429,53	2,01	2,16
22	C17	T2	Apex	Apex	I	567,51	2,07	2,32
29	C17	T2	Apex	Apex	II	471,88	1,99	2,19
36	C17	T2	Apex	Apex	III	397,63	2,02	2,24
43	C18	V1	Apex	Apex	I	397,84	2,01	2,23
50	C18	V1	Apex	Apex	II	387,35	2,02	2,26
57	C18	V1	Apex	Apex	III	419,9	2,01	2,24
64	C18	V2	Apex	Apex	I	370,49	2,02	2,28
73	C18	V2	Apex	Apex	II	470,44	1,98	2,21
82	C18	V2	Apex	Apex	III	316,12	2,03	2,22
2	C17	V1	Zone A	bud	I	440,24	2	2,13
3	C17	V1	Zone A	stem	I	337,37	2,03	2,17
9	C17	V1	Zone A	bud	II	362,99	2,02	2,15
10	C17	V1	Zone A	stem	II	272,89	2,02	2,14
16	C17	V1	Zone A	bud	III	267,62	2,02	2,16
17	C17	V1	Zone A	stem	III	282,37	2,02	2,14
23	C17	T2	Zone A	bud	I	331,62	2,05	2,27
24	C17	T2	Zone A	stem	I	310,61	2,06	2,3
30	C17	T2	Zone A	bud	II	634,03	2,09	2,31
31	C17	T2	Zone A	stem	II	392,75	2,02	2,24
37	C17	T2	Zone A	bud	III	668,89	2,07	2,29
38	C17	T2	Zone A	stem	III	408,54	2,01	2,23
44	C18	V1	Zone A	bud	I	345,26	2,01	2,27
45	C18	V1	Zone A	stem	I	344,36	2,04	2,27
51	C18	V1	Zone A	bud	II	398,78	2,02	2,25
52	C18	V1	Zone A	stem	II	389,27	2,02	2,25
58	C18	V1	Zone A	bud	III	273,79	2,06	2,28
59	C18	V1	Zone A	stem	III	265,54	2,05	2,29
65	C18	V2	Zone A	bud	I	242,13	2,05	2,31
66	C18	V2	Zone A	stem	I	262,36	2,05	2,3
74	C18	V2	Zone A	bud	II	153,28	2,03	2,29
75	C18	V2	Zone A	stem	II	313,68	2,05	2,18
83	C18	V2	Zone A	bud	III	303,1	2,03	2,26
84	C18	V2	Zone A	stem	III	300,33	2,03	2,3
4	C17	V1	Zone B	bud	I	199,07	2,03	2,06
5	C17	V1	Zone B	stem	I	170,65	2	2,01
11	C17	V1	Zone B	bud	II	196,13	2,03	2,1
12	C17	V1	Zone B	stem	II	200,24	2,04	2,1
18	C17	V1	Zone B	bud	III	251,95	2,02	2,06
19	C17	V1	Zone B	stem	III	109,18	2,11	2,29
25	C17	T2	Zone B	bud	I	359,1	2,03	2,28
26	C17	T2	Zone B	stem	I	224,68	2,06	2,3
32	C17	T2	Zone B	bud	II	442,18	2	2,24

33	C17	T2	Zone B	stem	II	335,39	2,04	2,27
39	C17	T2	Zone B	bud	III	572,54	2,06	2,25
40	C17	T2	Zone B	stem	III	250,5	2,07	2,3
46	C18	V1	Zone B	bud	I	303,23	2,05	2,28
47	C18	V1	Zone B	stem	I	209,91	2,06	2,28
53	C18	V1	Zone B	bud	II	139,92	2,05	2,16
54	C18	V1	Zone B	stem	II	253,49	2,08	2,34
60	C18	V1	Zone B	bud	III	186,16	2,09	2,29
61	C18	V1	Zone B	stem	III	271,56	2,03	2,28
67	C18	V2	Zone B'	bud	I	272,11	2,03	2,28
68	C18	V2	Zone B'	stem	I	170,57	2,06	2,27
76	C18	V2	Zone B'	bud	II	239,78	2,06	2,31
77	C18	V2	Zone B'	stem	II	183,99	2,07	2,29
85	C18	V2	Zone B'	bud	III	333,45	2,03	2,21
86	C18	V2	Zone B'	stem	III	194,87	2,05	2,3
69	C18	V2	Zone B''	bud	I	226,65	2,05	2,27
70	C18	V2	Zone B''	stem	I	114,68	2,05	2,25
78	C18	V2	Zone B''	bud	II	187,66	2,05	2,28
79	C18	V2	Zone B''	stem	II	117,97	2,06	2,26
87	C18	V2	Zone B''	bud	III	255,6	2,05	2,29
88	C18	V2	Zone B''	stem	III	129,14	2,04	2,27
6	C17	V1	Zone C	bud	I	263,48	2,03	2,13
7	C17	V1	Zone C	stem	I	190,56	2,03	2,07
13	C17	V1	Zone C	bud	II	184,2	2,03	2,1
14	C17	V1	Zone C	stem	II	210,54	2,03	2,09
20	C17	V1	Zone C	bud	III	112,22	2,08	2,34
21	C17	V1	Zone C	stem	III	199,39	2,09	2,3
27	C17	T2	Zone C	bud	I	252,68	2,05	2,28
28	C17	T2	Zone C	stem	I	218,94	2,05	2,22
34	C17	T2	Zone C	bud	II	340,64	2,02	2,25
35	C17	T2	Zone C	stem	II	257,89	2,05	2,23
41	C17	T2	Zone C	bud	III	311,18	2,03	2,28
42	C17	T2	Zone C	stem	III	264,25	2,06	2,3
48	C18	V1	Zone C	bud	I	165,75	2,09	2,32
49	C18	V1	Zone C	stem	I	120,59	2,06	2,25
55	C18	V1	Zone C	bud	II	191,95	2,09	2,28
56	C18	V1	Zone C	stem	II	166,55	2,06	2,26
62	C18	V1	Zone C	bud	III	152,85	2,09	2,27
63	C18	V1	Zone C	stem	III	145,65	2,05	2,27
71	C18	V2	Zone C	bud	I	118,07	2,06	2,19
72	C18	V2	Zone C	stem	I	101,32	2,11	2,35
80	C18	V2	Zone C	bud	II	132,28	2,09	2,32
81	C18	V2	Zone C	stem	II	82,43	2,13	2,2
89	C18	V2	Zone C	bud	III	185,87	2,05	2,3
90	C18	V2	Zone C	stem	III	86,31	2,07	2,26
