# Supplementary material for: Change in Auxin and Cytokinin Levels Coincides with Altered Expression of Branching Genes during Axillary Bud Outgrowth in Chrysanthemum
Source: PLoS One. 2016 Aug 24;11(8):e0161732. doi: 10.1371/journal.pone.0161732 (PMC4996534; doi:10.1371/journal.pone.0161732)
Supplement: S4 Table — (PDF) [file pone.0161732.s008.pdf]

Type	Gene	Forward primer	Reverse primer	Forward Tm (C°)	Reverse Tm (C°)	Amplicon length (bp)
Reference genes	***CmUBC***	ATGGACCGCACTTATCAAGG	AGGAGGCTGCAAAGGGTATT	54,8	56,5	100
	CmUBQ10	CGAGAGCTCTGACACCATTG	CGTCCTCGAGCTGCTTTC	55,5	56	107
	CmEF1α	ACACCAAGGGTGAAAGCAAG	GGCTGATTGTGCTGTCTTGA	55,8	55,5	111
	CmACT2	GACCTTCAATGTTCCGGCTA	CACACCATCACCAGAATCCA	54,8	54,8	103
	CmATUB	CCAGATGCCAAGTGACAAAA	GCGGTAAGTTCCAGTCCTCA	53,5	56,7	151
	CmCACS	ATACCGTTGAGTGGGTCAGG	GGGTCACCATGTCATCAAGA	56,6	54,5	103
	CmEXP5	GCAGTCGGATTTTCGTCACT	GCGGGTTATGTGCGATATGT	55,4	55,4	104
	CmEXP6	ACCCCTATGCACAAGGTGTC	TAGCACATGCAGTCGGATTT	57,2	54,5	150
	CmPGK	AAAGAGGCCATTTGCTGCTA	TCCAATCTTGGATGAGACCTTT	54,8	54,9	92
	CmPSAA	GAAGCCCAGACAAAATGAGC	GGCACAGTCCTCCCAAGTAA	54,3	56,8	90
	CmBTUB	TGTCGGATACCGTTGTTGAG	AGTCGCCAAAGCTTGGATTA	54,6	54,5	150
	CmMTP	AAGCCACAAGCTAACCCTGA	GAAGCTCTCCTGCCATCAAC	56,6	55,9	152
	CmHH3	TCACCGAAAATGAAATGCAA	TGAGGCTTACCTGGTTGGTC	50,9	56,8	160
Target genes	***CmBRC1***	TGCTGTGTCGGTCTTTCTTG	AATGTGCAGTCCACCATGAC	55,3	55,6	114
	CmIPT3	ACCCCGCCGAAATAATAAAT	CCCTCTTCCCACTACCGATT	52,1	54,3	121
	CmLSL	ACCTCGCCGGAAGAGTTTAC	CACATTTTCAGTCGCTCCAA	57	53,4	114
	CmMAX1	CAGAGCCGGAAAAGTTCAAG	GAGGTCCAATCCCAAATGGT	53,9	54,9	99
	CmMAX2	CGACTACACGAATCTGCTCTC	CACTTTTTGATGTTTCTTTTGAGG	54,9	51,3	119
	CmMAX3	ACCATAAAACCGGTGAGTGG	CAACCTATCGCCCCAACTTA	55	54,6	134
	CmDRM1	GATGGTGATGGTGTTGTTGG	ACCATGTCGGAAGGTGAAGG	54,1	57,1	98
	CmPIN1	ACAGCAGTGGTGCCATTGTA	TGCAAAAAGTGCAACAAAGC	57,1	53,2	117
	CmAXR1	GGGCAAAACTGGAGAATTGA	AGAAGATCGGCAGAGATCCA	53,3	55,3	128
	CmTIR1	TGCCAATAACCGTCCTAACC	ACGTTGGAGTCCTTTGCAGT	54,8	56,9	133
	CmIAA12	GGAAAAACAGCAACGCTCTC	CCGTGCTCAGAAACATCCTC	54,7	55,8	122
	CmRR1	CAGGATGTCGGTTTGACCTT	GGAAGGACGGTTATGCTGAA	55	54,8	99
	CmHK3a	TCCTTGCCGGTCAACATATC	CTTCGCCCATTCAACAAGA	54,8	53	92
	CmHK3b	TCCTTGCCGGTCAACATATC	AAAGAAGATGACCCCCGTAGA	54,8	55,3	144
	CmSTM	GCCTAGTCCACCAGTCAAGC	GATCGGGAACTAAAGGGTCA	57,9	54	139
	CmTIR3	CCGTCCCAGTTCTTCTCTGA	GGCCCAGCTTCATTACAAAA	56,2	53,6	101
	CmAXR2	CAAGAACGTGATGGCTCAAA	GGTCCACCTTGCGAAGATAA	54,5	53,4	134
	CmIAA16	AACGCACTTGACCATCTCTG	TTGGCAAAGGAAAACCAATC	51,7	53,3	159
	CmAXR6	GCAAATTGATGGACCTTTTGA	CCCAATTGCTTCCTTTCCTT	55,2	51,7	152
