# Supplementary material for: Change in Auxin and Cytokinin Levels Coincides with Altered Expression of Branching Genes during Axillary Bud Outgrowth in Chrysanthemum
Source: PLoS One. 2016 Aug 24;11(8):e0161732. doi: 10.1371/journal.pone.0161732 (PMC4996534; doi:10.1371/journal.pone.0161732)
Supplement: S5 Table — In batch 1 the expression of CmBRC1, CmIPT, CmLsL, CmMAX1 and CmMAX2 was analysed. Batch 2 was used for expression analysis of CmRR1, CmAXR1, CmAXR2, CmAXR6, CmHK3a, CmHK3b, CmDRM1, CmIAA12, CmIAA16, CmMAX3, CmPIN1, CmSTM, CmTIR1 and CmTIR3. (PDF) [file pone.0161732.s009.pdf]

	Shoot apex/Bud			Stem		
**Type**	**Gene**	**PCR efficiency**		**Gene**	**PCR efficiency**	
		Batch1	Batch 2		Batch 1	Batch2
Reference genes	***CmUBC***	1,93	1,92	***CmACT2***	1,93	1,92
	CmATUB	1,91	1,92	***CmATUB***	1,91	1,91
	CmEF1α	1,89	1,89	***CmUBQ10***	1,90	1,89
Target genes	***CmBRC1***	1,92		***CmBRC1***	1,91	
	CmDRM1		1,92	***CmDRM1***		1,91
	CmLsL	1,86		***CmLSL***	1,85	
	CmSTM		1,92	***CmSTM***		1,92
	CmMAX1	1,90		***CmMAX1***	1,91	
	CmMAX2	1,89		***CmMAX2***	1,87	
	CmMAX3		1,88	***CmMAX3***		1,88
	CmIPT3	1,84		***CmIPT3***	1,85	
	CmRR1		1,92	***CmRR1***		1,90
	CmHK3 a		1,90	***CmHK3 a***		1,89
	CmHK3 b		1,91	***CmHK3 b***		1,90
	CmAXR1		1,89	***CmAXR1***		1,92
	CmAXR2		1,86	***CmAXR2***		1,87
	CmAXR6		1,88	***CmAXR3***		1,86
	CmIAA16		1,90	***CmAXR6***		1,90
	CmIAA12		1,91	***CmIAA12***		1,89
	CmPIN1		1,85	***CmPIN1***		1,89
	CmTIR1		1,91	***CmTIR1***		1,90
	CmTIR3		1,91	***CmTIR3***		1,90
