# Supplementary material for: Change in Auxin and Cytokinin Levels Coincides with Altered Expression of Branching Genes during Axillary Bud Outgrowth in Chrysanthemum
Source: PLoS One. 2016 Aug 24;11(8):e0161732. doi: 10.1371/journal.pone.0161732 (PMC4996534; doi:10.1371/journal.pone.0161732)
Supplement: S6 Table — M and CV values for the 3 reference genes that were used for normalisation in the RT-qPCR analysis of branching genes in apex and axillary bud tissue and in stem samples. (PDF) [file pone.0161732.s010.pdf]

Tissue	Reference gene	Batch 1		Batch 2	
		M	CV	M	CV
Apex/Axillary buds	*CmATUB*	0.608	0.275	0.607	0.266
	CmUBC	0.526	0.218	0.56	0.258
	CmEF1α	0.516	0.199	0.472	0.144
	Mean	0.55	0.231	0.546	0.223
Stem	*CmACT2*	0.33	0.103	0.327	0.089
	CmATUB	0.42	0.179	0.458	0.2
	CmUBQ10	0.389	0.165	0.402	0.179
	Mean	0.379	0.149	0.396	0.156
