# Supplementary material for: Change in Auxin and Cytokinin Levels Coincides with Altered Expression of Branching Genes during Axillary Bud Outgrowth in Chrysanthemum
Source: PLoS One. 2016 Aug 24;11(8):e0161732. doi: 10.1371/journal.pone.0161732 (PMC4996534; doi:10.1371/journal.pone.0161732)
Supplement: S8 Table — Data are fold changes of hormone content (A-B = Zone-B/Zone-A) between mean (n = 3) IAA or CK contents and the significant difference between means by Kruskal-Wallis test is indicated by * (p-value<0.05). (PDF) [file pone.0161732.s012.pdf]

C17		Bud			Stem		
		A-B	A-C	B-C	A-B	A-C	B-C
V1	IAA	-4.9*	-5.5*	-1.1	1.1	1.1	1.1
	CK	-4.5*	-24.2*	-5.4*	-1.3*	1.9*	2.5*
T2	IAA	-2.1	-1.5	1.4	-1.7	6.2	10.4*
	CK	-3.5*	-13.4*	-3.8	-2.9	-9*	-3.1

C18		Bud			Stem		
		A-B	A-C	B-C	A-B	A-C	B-C
V1	IAA	-1.8	-3.8	-2.1	-1.5	-1.6	-1.1
	CK	-1.5	-3.2	-2.2	-1.4	-1.2	1.2

		Bud						Stem					
		A-B'	A-B''	A-C	B'-B''	B'-C	B''-C	A-B'	A-B''	A-C	B'-B''	B'-C	B''-C
V2	IAA	-1.3	-2.3	-3.6	-1.8	-2.8	-1.6	-1.0	-1.2	-1.7	-1.2	-1.6*	-1.4*
	CK	-2.9	-4.1	-10.7	-1.4	-3.7	-2.6	2.6	2.2	1.4	-1.2	-1.9	-1.6
