# Supplementary material for: Change in Auxin and Cytokinin Levels Coincides with Altered Expression of Branching Genes during Axillary Bud Outgrowth in Chrysanthemum
Source: PLoS One. 2016 Aug 24;11(8):e0161732. doi: 10.1371/journal.pone.0161732 (PMC4996534; doi:10.1371/journal.pone.0161732)
Supplement: S9 Table — Data are fold changes (A-B = Zone-B/Zone-A) between mean (n = 3) IAA or CK contents and the significant difference between means by Kruskal-Wallis test is indicated by * (p-value<0.05). (PDF) [file pone.0161732.s013.pdf]

C17	Bud			Stem		
	A-B	A-C	B-C	A-B	A-C	B-C
IAA	-3.6*	-2.5	1.9	-13.3*	-1.3	-1.4
CK	-2.0*	-7.5*	-1.7	-1.1	-3.4*	-2.5*

C18	Bud				Stem			
	A-B'	A-B''	A-C	B-C	A-B'	A-B''	A-C	B-C
IAA	-1.1	-2.1	-3.2*	-1.8	-1.8	-2.1	-2.9*	-2.0*
CK	-6.6*	-9.2	-24.1	-16.4	-2.7	-3.2	-5.1	-3.7
