# Supplementary material for: Change in Auxin and Cytokinin Levels Coincides with Altered Expression of Branching Genes during Axillary Bud Outgrowth in Chrysanthemum
Source: PLoS One. 2016 Aug 24;11(8):e0161732. doi: 10.1371/journal.pone.0161732 (PMC4996534; doi:10.1371/journal.pone.0161732)
Supplement: S11 Table — Data are fold changes (A-B = Zone-B/Zone-A) between mean CNRQ values (n = 3). The significant difference between means by Kruskal-Wallis test is indicated by * (p-value<0.05). (PDF) [file pone.0161732.s015.pdf]

		V1						T2					
		Apex-A	Apex-B	Apex-C	A-B	A-C	B-C	Apex-A	Apex-B	Apex-C	A-B	A-C	B-C
Bud dev.	*CmBRC1*	4,8*	8*	7,1*	1,7	1,5	-1,1	4*	7,8*	15,9*	1,9	4*	2*
	CmDRM1	1,5	9,2*	5,7*	6,2	3,8*	-1,6	1,1	-1,1	7,2*	-1,2	7*	8,2
	CmLsL	1,2	1,6*	1,2	1,4	-1	-1,4*	-1,3*	1	2,2*	1,3*	2,8*	2,2*
	CmSTM	2,2*	1,1	-1,2	-2*	-2,7*	-1,4	1,5*	1,1	1,2	-1,3	-1,3*	1
	CmMAX1	1,1	1,9	1,5	1,8*	1,5*	-1,2	1,1	1,3*	2*	1,2	1,8*	1,5*
SL	*CmMAX2*	-1,3	1	-1,4	1,4	-1,1	-1,4	-1,6	-1,4*	-1	1,1	1,5	1,4
	CmIPT3	-1	25,4*	21,1*	25,6*	21,3	-1,2*	1,4	3	44,7*	2,2	32,8*	14,7*
	CmRR1	-1,2	1,7*	2,5*	2,1*	3*	1,4	-1,4*	1,1	1,8	1,6	2,6*	1,7
	CmHK3 a	1,1	1,5*	1,9*	1,4	1,7	1,2	-1,8*	-1,2	-1,2	1,5	1,5	-1
CK	*CmHK3 b*	-1	1,8*	1,7*	1,9*	1,7*	-1,1	-1,6	-1,1	-1	1,5	1,6	1,1
	CmPIN1	1,3*	-1,8*	-2,5*	-2,3*	-3,3*	-1,4*	1,1	1,2	-1,7*	1,1	-1,7*	-2
AUX trans.	*CmTIR3*	-1	1,9*	2,8	1,9	2,8	1,5	-1,3	1,6	2,2	2,1	2,5	1,3
	CmTIR1	1	2*	1,6*	2*	1,6*	-1,2	-1,1	-1,1	2,3*	1,1	2,6*	2,5
	CmAXR1	-1,1	1,4	1,3	1,6*	1,5	-1,1	1	1,2	1,3*	1,1	1,3	1,1
AUX sign.	*CmAXR6*	-2,1*	1,9*	1,3	4,1*	2,7*	-1,5*	-2,8*	-1,7	1,8*	1,6	5*	3,1
	CmAXR2	-2,2*	2,2*	1,9	5*	4,4*	-1,1	-3,2*	-2,2	1,9*	1,4	6*	4,2
	CmIAA16	-1,1	-1,1	1	1	1,2	1,1	-1,3	1	1	1,3	1,3	1
AUX resp.	*CmIAA12*	1,7*	-1,3	-1,8*	-2,1	-2,9	-1,4	1,3*	-1	-1,3	-1,4	-1,7*	-1,2
