# Supplementary material for: Change in Auxin and Cytokinin Levels Coincides with Altered Expression of Branching Genes during Axillary Bud Outgrowth in Chrysanthemum
Source: PLoS One. 2016 Aug 24;11(8):e0161732. doi: 10.1371/journal.pone.0161732 (PMC4996534; doi:10.1371/journal.pone.0161732)
Supplement: S12 Table — Data are fold changes (A-B = Zone-B/Zone-A) between mean CNRQ values (n = 3). The significant difference between means by Kruskal-Wallis test is indicated by * (p-value<0.05). (PDF) [file pone.0161732.s016.pdf]

		V1			T2		
		A-B	A-C	B-C	A-B	A-C	B-C
Bud dev.	*CmBRC1*	2,3*	1,9	-1,2	1,4	2,3*	1,7*
	CmDRM1	3,4	4,6*	1,4	-1	3*	3,1*
	CmLsL	1,2	1	-1,2	1,2	1	-1,2
	CmSTM	-1,6*	-1,8*	-1,1	-1,7*	-2,4*	-1,5*
SL	*CmMAX1*	1	-1	-1	1,1	-1	-1,1
	CmMAX2	1,2	1,9	1,6	-2,2*	-4,2	-1,9
CK	*CmIPT3*	2,5*	1,7	-1,5	1,9	4,9*	2,5*
	CmRR1	1,2	1,8*	1,5*	-1,5	-2,1*	-1,4
	CmHK3 a	2,4*	2,5*	1	-1,2	1,2	1,4*
	CmHK3 b	2,2*	2,3*	1	-1,3	-1	1,2
AUX trans.	*CmPIN1*	-1,4*	-1,1	1,3*	-1,3	-2*	-1,6*
	CmTIR3	2,6*	2*	-1,3	1,2	1,6	1,3
AUX sign.	*CmTIR1*	1,7*	1,6*	-1,1	-1,1	-1,1	-1
	CmAXR1	-1	1	1,1	-1,1*	-1,3*	-1,2
	CmAXR6	-1,2	-1,3*	-1,1	-1	-1,4*	-1,3*
AUX resp.	*CmAXR2*	-1,6	-1,6*	-1	-1	-1,5*	-1,5*
	CmIAA16	-1,1	1,1*	1,2*	-1,5*	-1,6*	-1,1
	CmIAA12	1,1	1,1	1,1	1,1	-1,1	-1,2
