# Supplementary material for: Change in Auxin and Cytokinin Levels Coincides with Altered Expression of Branching Genes during Axillary Bud Outgrowth in Chrysanthemum
Source: PLoS One. 2016 Aug 24;11(8):e0161732. doi: 10.1371/journal.pone.0161732 (PMC4996534; doi:10.1371/journal.pone.0161732)
Supplement: S13 Table — Data are fold changes (A-B = Zone-B/Zone-A) between mean CNRQ values (n = 3). The significant difference between means by Kruskal-Wallis test is indicated by * (p-value<0.05). (PDF) [file pone.0161732.s017.pdf]

		Bud						Stem					
		A-B	A-C	B-C	B-A	C-A	C-B	A-B	A-C	B-C	B-A	C-A	C-B
Bud dev.	*CmBRC1*	1,4	1,69	2,8*	-2,3*	-2*	-1,06	-1,42	1,17	-4,6*	-2*	-3,8*	-2,7*
	CmDRM1	-1,14	-3,28	1,9	-22,9*	-14,1*	-4,38	-3,54	-1,13	-11,36	-3,8*	-15,6*	-16,3*
	CmLsL	-1,18	1,37	1,9*	-2	-1,5	-1,15	-1,02	-1,2*	-1,5*	-1,4*	-1,2*	-1,06
	CmSTM	-1,13	1,8*	-1,09	2,3*	3,1*	2,39	-1,15	-1,7*	2,34	-1,1*	2,5*	1,5*
	CmMAX1	-1,05	-1,21	1,4*	-2,1*	-1,8*	-1,5*	-1,2*	-1,3*	-1,3*	-1,34	-1,26	-1,17
SL	*CmMAX2*	1,8*	1,9*	2,6*	1,2	1,74	1,9*	1,39	-1,4*	2,59	-1,6	1,62	-1,35
	CmIPT3	-1,44	-2,5*	10,2*	-82,2*	-68,3*	-30,6*	-1,8*	1,44	-8,4*	-1,7*	-5,7	-3*
	CmRR1	1,64	1,21	2,5*	-2,1*	-3*	-1,81	1,06	-1,33	1,3*	-1,58	-1,13	-1,7*
	CmHK3 a	1,34	1,45	-1,05	-1,03	-1,26	1,67	1,01	1,5*	-1,9*	-1,7	-2*	-2,5*
CK	*CmHK3 b*	2,32	1,3	2,5*	-1,25	-1,14	1,34	1,02	1,26	-1,65	-1,72	-1,7*	-2,2*
	CmPIN1	1,23	1,7*	-1,37	3*	4,3*	4,04	1,03	-1,5*	1,81	-1,1*	1,38	1,1
AUX trans.	*CmTIR3*	1,46	1,23	1,78	-2*	-2,72	-1,3	1,41	1,9*	-2,24	-1,4*	-1,69	-1,41
	CmTIR1	1,54	1,4*	2,7*	-1,9*	-1,5*	-1,03	1,15	1,2	-1,3*	-1,4*	-1,21	-1,3*
	CmAXR1	1,39	-1,13	1,4*	-1,5*	-1,44	-1,11	-1,1	-1,3*	1,1*	-1,3	-1,01	-1,2*
AUX sign.	*CmAXR6*	2,02	-1,02	4*	-5*	-3,4*	-1,34	-1,05	-1,5*	1,19	-1,17	1,3*	1,3*
	CmAXR2	1,71	1,03	5,1*	-5,8*	-5*	-2,54	-1,12	-1,7*	1,45	-1,1*	-1,5*	1,5*
	CmIAA16	1,48	1,5	1,6*	1,1*	-1,02	1,25	-1,11	-1,2*	1,42	-1,1*	1,17	-1,3*
AUX resp.	*CmIAA12*	-1,27	1,37	-1,5*	2,3*	3,1*	3,14	-1,06	-1,3*	-1,27	-1,35	-1,37	-1,22
