# Supplementary material for: Change in Auxin and Cytokinin Levels Coincides with Altered Expression of Branching Genes during Axillary Bud Outgrowth in Chrysanthemum
Source: PLoS One. 2016 Aug 24;11(8):e0161732. doi: 10.1371/journal.pone.0161732 (PMC4996534; doi:10.1371/journal.pone.0161732)
Supplement: S14 Table — Data are fold changes (A-B = Zone-B/Zone-A) between mean CNRQ values (n = 3). The significant difference between means by Kruskal-Wallis test is indicated by * (p-value<0.05). (PDF) [file pone.0161732.s018.pdf]

		V1						V2									
		Apex-A	Apex-B	Apex-C	A-B	A-C	B-C	Apex-A	Apex-B'	Apex-B'	Apex-C	A-B'	A-B''	A-C	B'-B''	B'-C	B''-C
Bud dev.	*CmBRC1*	5,5*	11,4*	6,4*	2,1*	1,15	-1,8*	6,2*	13,2*	11,5*	5,4*	2,1*	1,8*	-1,16	-1,15	-2,5*	-2,1*
	CmDRM1	6,29	17,2*	22,2*	2,73	3,54	1,29	6,1*	12,1*	9*	7,3*	2*	1,47	1,18	-1,35	1,7*	-1,24
	CmLsL	1,22	2*	2*	1,7*	1,6*	-1,04	2*	3,3*	3,1*	3,6*	1,6*	1,6*	1,8*	-1,05	1,1	1,15
	CmSTM	3,8*	3*	-1,02	-1,25	-3,9	-3,1	2,5*	6*	4,8*	1,5*	2,4*	1,9*	-1,6*	-1,24	-3,9*	-3,2*
SL	*CmMAX1*	2*	2,8*	2,5*	1,4	1,2	-1,13	3*	3*	2,4*	2,5*	1	-1,3*	-1,19	-1,28	-1,19	1,08
	CmMAX2	1,77	3*	1,9*	1,74	1,05	-1,66	3,4*	3,3*	1,9*	1,26	-1,02	-1,8*	-2,7*	-1,8*	-2,6*	-1,5*
CK	*CmIPT3*	1,67	4,1*	66*	2,45	40*	16*	-1,01	2,5*	8,9*	30,6*	1,43	5,18	17,79	3,6*	12,5*	3,4*
	CmRR1	1,46	1,63	3*	1,12	2,1*	1,85	2,3*	3,5*	4,1*	30,7*	1,5*	1,8*	1,8*	1,17	1,2*	1,02
	CmHK3 a	3*	2,3*	1,7*	-1,31	-1,8*	-1,33	2,5*	4,3*	2,9*	1,6*	1,7*	1,16	-1,6*	-1,5*	-2,8*	-1,83
	CmHK3 b	1,7*	2*	1,9*	1,19	1,14	-1,04	2,8*	2,8*	2*	1,8*	1,02	-1,3*	-1,6	-1,4*	-1,6	-1,17
AUX trans.	*CmPIN1*	1,5*	-1,06	-3,5*	-1,6*	-5,2*	-3,3*	1,7*	1,15	-1,16	-3,1*	-1,5*	-2*	-5,4*	-1,33	-3,6*	-2,7*
	CmTIR3	-1,5*	1,05	1,08	1,64	1,7	1,03	1,8*	1,43	1,43	2,3*	-1,26	-1,26	1,31	1	1,65	1,65
AUX sign.	*CmTIR1*	1,18	3,8*	5,4*	3,2*	4,6*	1,43	1,7*	2,8*	3*	3,5*	1,62	1,74	2*	1,07	1,26	1,17
	CmAXR1	-1,07	1,3	1,88	1,38	2,01	1,45	1,6*	1,8*	2*	2,4*	1,1	1,3*	1,5*	1,1*	1,4*	1,19
	CmAXR6	-1,05	1,37	5,1*	1,45	5,4*	3,7*	-1,3*	2,7*	2,9*	3,4*	3,6*	3,9*	4,6*	1,09	1,3*	1,19
AUX resp.	*CmAXR2*	-1,15	1,41	11,1*	1,63	13*	7,9*	-1,7*	2,9*	4,54	7,3*	4,9*	7,7	12,4*	1,59	2,6*	1,61
	CmIAA16	-1,02	1,1	1,4*	1,13	1,4*	1,28	1,7*	1,7*	1,6*	1,4*	1,02	-1,05	-1,2*	-1,07	-1,2*	-1,16
	CmIAA12	1,1*	-1,3*	-5,2*	-1,5*	-5,9*	-4*	1,08	-1,4*	-1,5*	-4,4*	-1,5*	-1,7	-4,8	-1,08	-3*	-2,9*
