# Supplementary material for: Change in Auxin and Cytokinin Levels Coincides with Altered Expression of Branching Genes during Axillary Bud Outgrowth in Chrysanthemum
Source: PLoS One. 2016 Aug 24;11(8):e0161732. doi: 10.1371/journal.pone.0161732 (PMC4996534; doi:10.1371/journal.pone.0161732)
Supplement: S15 Table — Data are fold changes (A-B = Zone-B/Zone-A) between mean CNRQ values (n = 3). The significant difference between means by Kruskal-Wallis test is indicated by * (p-value<0.05). (PDF) [file pone.0161732.s019.pdf]

		V1			V2				
		A-B	A-C	B-C	A-B'	A-B''	A-C	B'-C	B''-C
Bud dev.	*CmBRC1*	1,8*	1,64	-1,12	1,7*	1,68	2,4*	1,42	1,43
	CmDRM1	1,34	2,16	1,61	1,07	2,7*	3,2*	3*	1,18
	CmLsL	1,1*	1,1*	-1	1,03	1,1*	1,5*	1,4*	1,3*
	CmSTM	-1,52	-1,4*	1,05	-2,1*	-2,2*	-2,4*	-1,1	-1,09
	CmMAX1	1,01	1,47	1,46	-1,16	1,3*	1,3*	1,6*	1,02
SL	*CmMAX2*	-1,17	-1,1*	1,1*	-1,39	-1,12	1,19	1,6*	1,33
	CmIPT3	1,94	3,93	2,03	2,5*	9,8*	16,6*	6,7*	1,7*
	CmRR1	-1,1*	1,3*	1,52	-1,4*	1,13	1,4*	1,96	1,2
	CmHK3 a	-1,6	-1,46	1,09	-2,6*	-2,1	-2,7	-1,07	-1,3*
CK	*CmHK3 b*	-1,24	-1,1*	1,2*	-1,8*	-1,32	-1,4	1,3*	-1,07
	CmPIN1	-1,3*	-1,3*	1,1*	-1,6*	-1,6*	-1,6*	-1,01	-1,01
AUX trans.	*CmTIR3*	2,42	3,64	1,5	2,2*	4,3*	4,9*	2,2*	1,14
	CmTIR1	1,3*	1,7*	1,28	1,43	2*	3*	2,1*	1,5*
	CmAXR1	-1,1*	1,3*	1,3*	-1,07	1,4*	1,4*	1,5*	1,05
AUX sign.	*CmAXR6*	-1,63	-1,8*	-1,12	-1,7*	-2,1*	-2,2*	-1,3*	-1,05
	CmAXR2	-1,5*	-2,1*	-1,4*	-1,6*	-1,7*	-2,1*	-1,3*	-1,3*
	CmIAA16	1,1*	1,17	1,1*	-1,3*	-1,09	-1,17	1,2*	-1,1*
AUX resp.	*CmIAA12*	-1,39	1,05	1,47	-1,8	-1,25	-1,2*	1,5*	-1
