# Supplementary material for: Change in Auxin and Cytokinin Levels Coincides with Altered Expression of Branching Genes during Axillary Bud Outgrowth in Chrysanthemum
Source: PLoS One. 2016 Aug 24;11(8):e0161732. doi: 10.1371/journal.pone.0161732 (PMC4996534; doi:10.1371/journal.pone.0161732)
Supplement: S16 Table — Data are fold changes (A-B = Zone-B/Zone-A) between mean CNRQ values (n = 3). The significant difference between means by Kruskal-Wallis test is indicated by * (p-value<0.05). (PDF) [file pone.0161732.s020.pdf]

		Bud				Stem			
		A-B'	A-B''	A-C	B-C	A-B'	A-B''	A-C	B-C
Bud dev.	*CmBRC1*	4,2*	3,7*	1,7*	-1,2	1,36	-1,36	1,3*	1,05
	CmDRM1	6,5*	4,8*	3,9*	1,42	2,18	1,6*	5,5*	4,8*
	CmLsL	1,9*	1,9*	2,1*	1,3*	1,04	-1,1*	1,2*	1,4*
	CmSTM	1,9*	1,57	-2*	-1,61	-1,7*	-1,1*	-1,8*	-1,26
SL	*CmMAX1*	1,7*	1,4*	1,5*	1,08	1,1	1,1*	1,7*	1,7*
	CmMAX2	2,5*	1,44	-1,03	-1,8	1,27	1,5*	1,6*	2,5*
CK	*CmIPT3*	2,32	8,4*	29*	11,8*	-1,1	-2,1*	3,6*	3,1*
	CmRR1	2,5*	3*	3*	2,7*	-1,04	1,1*	1,6*	2,1*
	CmHK3 a	2,1*	1,38	-1,3*	-1,02	-1,5*	1,1	-1,2*	1,02
	CmHK3 b	2,2*	1,6*	1,37	1,16	-1,07	1,16	1,26	1,5*
AUX trans.	*CmPIN1*	-1,4*	-1,8*	-4,8*	-3,1*	-1,37	-1,08	-1,37	-1,1
	CmTIR3	2,4*	2,4*	3,9*	2,4*	2,3*	-1,1*	4,5*	2,1*
AUX sign.	*CmTIR1*	4,3*	4,6*	5,4*	1,69	1,4*	1,1*	2*	2,3*
	CmAXR1	1,7*	2*	2,4*	1,7*	-1,13	-1,1*	1,3*	1,4*
	CmAXR6	3,8*	4,1*	4,9*	3,4*	-1,11	1,5*	-1,3*	1,1*
AUX resp.	*CmAXR2*	4,9*	5,96	12,5*	7,6*	-1,02	1,48	-1,03	1,14
	CmIAA16	1,7*	1,6*	1,4*	1,25	-1,03	-1,1*	1,2*	1,1*
	CmIAA12	-1,3*	-1,4*	-4,1*	-2,8	-1,5*	-1,06	1	1,4*
